# Predicting the impact of combined therapies on myeloma cell growth using a hybrid multi-scale agent-based model

**DOI:** 10.18632/oncotarget.13831

**Published:** 2016-12-09

**Authors:** Zhiwei Ji, Jing Su, Dan Wu, Huiming Peng, Weiling Zhao, Brian Nlong Zhao, Xiaobo Zhou

**Affiliations:** ^1^ Division of Radiologic Sciences and Center for Bioinformatics and Systems Biology, Wake Forest School of Medicine, Medical Center Boulevard, Winston-Salem, NC, USA 27157

**Keywords:** agent-based model, ODE, modeling, multiple myeloma, immune

## Abstract

Multiple myeloma is a malignant still incurable plasma cell disorder. This is due to refractory disease relapse, immune impairment, and development of multi-drug resistance. The growth of malignant plasma cells is dependent on the bone marrow (BM) microenvironment and evasion of the host's anti-tumor immune response. Hence, we hypothesized that targeting tumor-stromal cell interaction and endogenous immune system in BM will potentially improve the response of multiple myeloma (MM). Therefore, we proposed a computational simulation of the myeloma development in the complicated microenvironment which includes immune cell components and bone marrow stromal cells and predicted the effects of combined treatment with multi-drugs on myeloma cell growth. We constructed a hybrid multi-scale agent-based model (HABM) that combines an ODE system and Agent-based model (ABM). The ODEs was used for modeling the dynamic changes of intracellular signal transductions and ABM for modeling the cell-cell interactions between stromal cells, tumor, and immune components in the BM. This model simulated myeloma growth in the bone marrow microenvironment and revealed the important role of immune system in this process. The predicted outcomes were consistent with the experimental observations from previous studies. Moreover, we applied this model to predict the treatment effects of three key therapeutic drugs used for MM, and found that the combination of these three drugs potentially suppress the growth of myeloma cells and reactivate the immune response. In summary, the proposed model may serve as a novel computational platform for simulating the formation of MM and evaluating the treatment response of MM to multiple drugs.

## INTRODUCTION

Multiple myeloma (MM), a B-cell neoplasm, is characterized by clonal expansion of plasma cells in the hematopoietic bone marrow (BM) and over-production of circulating monoclonal immunoglobulin [1, 2]. MM has long been used as a paradigmatic model for investigating the role of the microenvironment in blood cancers [3]. Bone marrow, the natural niche of multiple myeloma, provides a milieu of growth factors and cytokines for multiple myeloma cell survival and proliferation. Therefore, the bone marrow microenvironment greatly contributes to the resistance of myeloma against various therapies. Interaction of myeloma cells with bone marrow stromal cells (BMSCs) via some key factors (SDF-1, TGFβ, IFNγ, IL6, and TNFα, etc.), induces pleiotropic anti-apoptotic mechanisms, thereby rendering multiple myeloma cells resistant to established therapeutic regimens [4, 5].

Recent studies have shown that a small population of CD138-negative B cells with “side population” characteristics presents in multiple myeloma [6, 7]. This cell population has the ability to give rise to clonogenic growth *in vitro* and possess stem cell characteristics. These myeloma initiating (stem) cells (MICs) have shown higher resistance to chemotherapeutic agents [8]. Our previous studies demonstrated that 1) BMSCs stimulated the growth and expansion of MICs [9]; and 2) the enhanced colony-forming and self-renewal ability of MICs were regulated via the centralized role of SDF-1 (stromal cell-derived factor 1) [9, 10]. We also established an agent-based model using the Markov Chain Monte Carlo approach to simulate the effects of SDF-1-induced chemo-physical communications among MICs and BMSCs on myeloma cell growth and examine if the biophysical properties of myeloma niches are druggable with two representative drugs: AMD3100, and Bortezomib (BTZ) [11]. However, the resistance of myeloma to those drugs was not only attributed to the myeloma-BMSC interactions.

The immune system has been known to modulate tumor cell growth, and tumor development can promote immunosuppression. Conversely, immunosuppression may support tumor development [12, 13]. Multiple myeloma-induced immune paresis is mainly attributed to the impairment of T-cell (CD4^+^, and CD8^+^) activation and proliferation, which is mediated by myeloma cell-induced production of transforming growth factor (TGFβ) [3, 12, 14]. Currently, immunomodulatory drugs, such as Lenalidomide (LEN) and Thalidomide (Thal), have been used to overcome conventional drug resistance and improve patient outcomes in MM [14]. Importantly, IMiDs-induced stimulatory effects on effector T cell and inhibitory role on T regulatory cells (Tregs) have been demonstrated *in vivo* [15, 16]. However, the precise cellular targets and the exact molecular mechanism of actions of IMiDs in multiple myeloma remain unclear. In clinic, the combined therapy with BTZ and LEN for the treatment of MM is widely used and is favorable for the initial therapy, but the majority of patients (50–60%) continue to suffer relapses [17]. An insight into the interactions of myeloma cells with BMSCs and immune cells in bone marrow microenvironment will potentially improve our understanding of myeloma growth, immune tolerance, and drug resistance.

**Figure 1:**
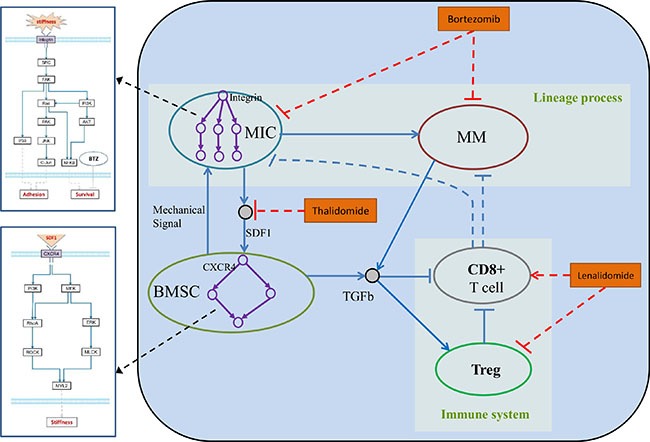
Hybrid multi-scale agent-based model of myeloma cell growth

Mathematical models have been used to simulate tumor growth or immune response in human [18, 19]. Everett, *et al*. proposed an approximation mathematical model of tumor growth and tumor-induced angiogenesis in ovarian cancer [20]. Eikenberry, *et al*. developed Partial Differential Equations (PDEs) to describe the basic process of melanoma invasion into healthy tissues [19]. However, systemic modeling of tumor growth (lineage process of cancer stem cell) and immune response within an integrated system was rarely studied. In this study, we proposed a 3D hybrid multi-scale agent-based model (HABM) to reveal the molecular mechanisms associated with myeloma development and immune escape in the bone marrow microenvironment (Figure 1). Hybrid model was composed of an ABM model and ODE system [43]. In our HABM, the ODEs were designed to simulate the dynamics of intracellular signal transductions and ABM to describe the cell-cell communications between stromal cells, tumor, and immune components in the Bone Marrow.

In the proposed model, we simulated the dynamics of MIC-derived myeloma development in a BM microenvironment to study the role of tumor-stroma interactions in MM progression and immune evasion. The BM microenvironment consisted of BMSCs and immune cells. This modeling system was classified into intracellular, intercellular, and tissue levels. The HABM model integrates events at different spatial and temporal scales. For the spatial scales, intracellular signaling pathways of BMSCs and MICs were simulated by ODEs to determine the biomechanical phenotype of BMSCs (cell stiffness) and tumor cell behaviors (such as migration and apoptosis). Cancer cells discern the changes of local stiffness of BSMCs and compete for the best location in the extracellular matrix (ECM) for their migration or proliferation. For the temporal scales, we modeled the intracellular signaling dynamics (minutes to hours); cell division, apoptosis, and local migration (hours to days); drug response (days to weeks); and tumor growth (weeks to months). In order to model the tumor growth and immune response of myeloma cells quantitatively within the same system, we mainly focused on the direct and indirect role of two important factors: SDF-1 and TGFβ. The studies from our groups and others have found that SDF-1α enhances the rigidity of BMSCs through its receptor CXCR4, and provides a proper environment for myeloma cell proliferation and migration [10, 11]. Immune tolerance of myeloma cells was mainly mediated by the production of TGFβ [21]. Our *in vitro* experiments also shown that SDF-1 and TGFβ play key roles in promoting the tumor growth, survival and propagation. SDF-1 triggers CXCR4 receptor dimerization and activate the intracellular signaling pathways of BMSCs, and the positive feedbacks from BMSC will change the behaviors of MICs. Secretion of TGFβ both from BMSCs and myeloma cells inhibited the proliferation of CD8^+^ T cells and promoted the expansion of Tregs. Moreover, activated Tregs suppressed the function of CD8^+^ T cells via induction of cell cycle arrest or apoptosis. Through the parameters tuning, the outcomes from our HABM model under different conditions were consistent with the experimental observations from previous studies. Moreover, to examine the potential targets of multiple myeloma in this microenvironment and discover novel therapeutic strategy, we further simulated the treatment effects of three representative drugs (BTZ, LEN and Thal). Our findings suggest that targeting SDF-1 and TGFβ in BM using a triple-combination with BTZ, LEN, and Thal, potentially improve the response of myeloma cells by increasing the inhibition of myeloma cell growth and activating the endogenous immune surveillance against tumor antigens.

In summary, the proposed HABM model provides new insight into the myeloma development in the bone marrow microenvironment carrying immune system; and also builds an efficient computational platform for prediction of drug response for discovering the optimal dose combination.

## RESULTS

### RPPA data analysis

**Figure 2:**
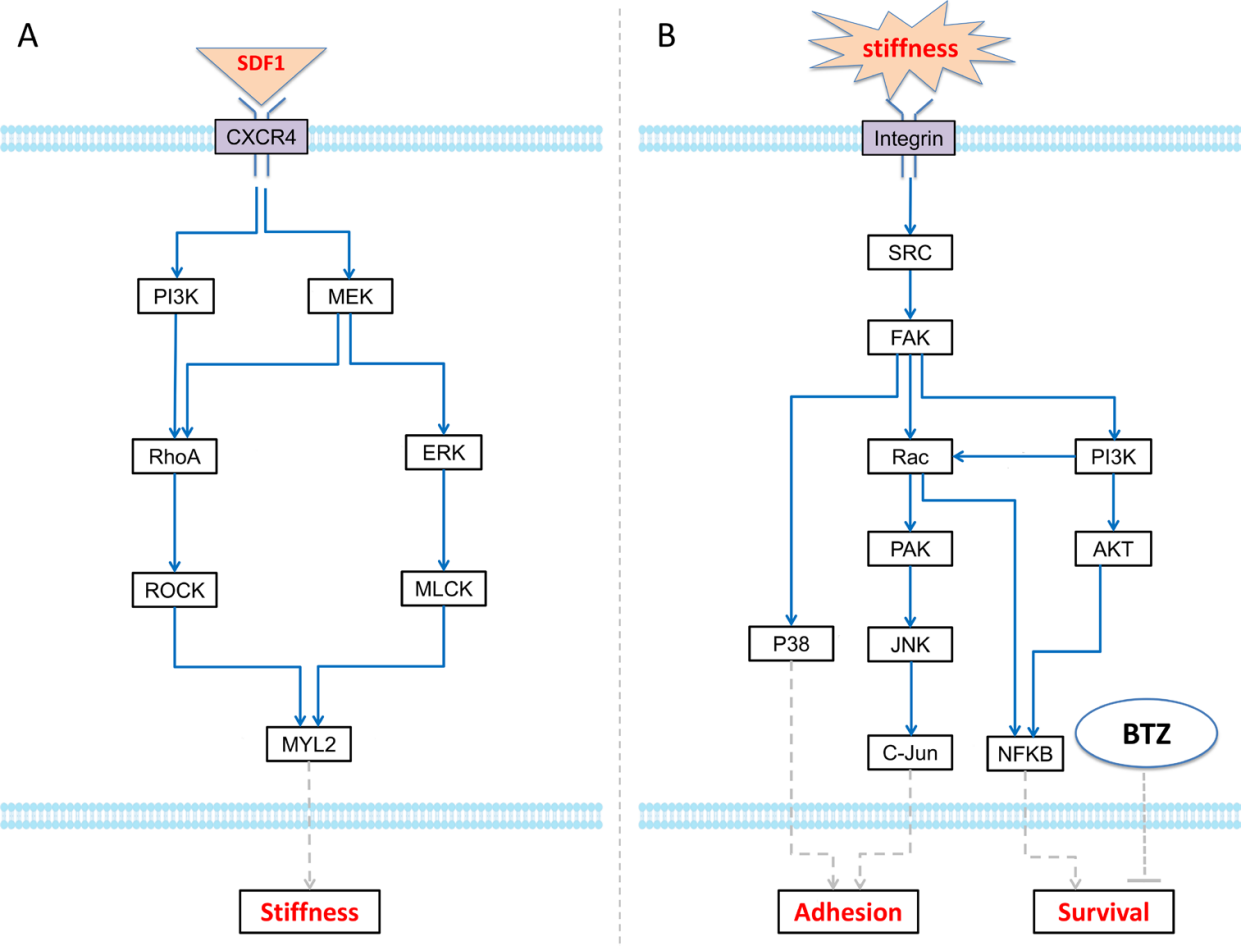
ODE systems in the proposed model (**A**) The intracellular signaling pathways in MIC cell, which modulates adhesion and survival rate to response the local BMSC stiffness; (B) The intracellular signaling pathways in BMSC cell, which determine the stiffness of BMSC section according to relative SDF-1 concentration.

In our previous studies, we have demonstrated that SDF-1α secreted by myeloma cells regulated the rigidity of BMSCs through binding to its receptor CXCR4, thereby, provided a proper environment for cell attachment, growth and migration [10]. Therefore, SDF-1α/CXCR4 axis appears to play an important role in myeloma cells-BMSC communication. Hence, we collected RPPA data to study the SDF-1-mediated signaling pathways. The myeloma BMSCs were treated with 100 ng/mL SDF-1α and then protein samples were collected at 0, 5, 10, and 15 min. The protein profiling was analyzed using Rreverse Phase Protein Array (RPPA) technology. There are 172 proteins in this RPPA dataset and the differentially expressed proteins were presented in the Supplementary Figure S1. However, stiffness-related signaling pathway was not well covered by our RPPA data. Choi, *et al*. proposed a model of biophysical regulation of BMSC. Binding of SDF-1 ligand to its cognate receptors CXCR4/CXCR7 results in the activation of PI3K or MEK. Subsequently, MYL2 is phosphorylated, which leads to changes in cell stiffness [10]. Hence, we did Western Blot for three key proteins (FAK, RhoA, and MYL2) as the complementary data [10, 22]. Focusing on the above measured proteins, we picked up a sub-graph from Choi's proposed network for simulating the dynamic changes of SDF-1-trigged BMSC stiffness, which in turn regulated the proliferation of MIC (Figure 2A).

To understand the effects of stiffness on myeloma cells, RPPA data were also collected in the myeloma cells treated with different stiffness (100Pa, and 400Pa) at 0, 30 min, 60 min, and 24 hour. The expression of an individual protein after treatment was normalized using the value obtained at *t* = 0. The samples were collected at 30–60 min and 24 hour represented the immediate and stable responses of MICs to different stiffness cues, respectively. Total 195 proteins were analyzed, in which 45 proteins up-regulated and 19 proteins down-regulated significantly. Several proteins were significantly upregulated under the condition of 400Pa, such as HSP70, BCL2, etc (Supplementary Table S1). Based on the measured proteins in the RPPA dataset and data collected from related literatures, we built an intracellular signaling pathway map for MIC (Supplementary Figure S2). Several important signaling pathways were included in this map. The stiffness of BMSC trigged the expression of FAK in MIC, which can increase cell adhesion via p38 and JNK signaling pathways, and promote cell survival via PI3K/AKT signaling. Moreover, autocrine SDF-1 signaling can also stimulate the expression of BCL2 and thereby inhibit the apoptosis of MIC by reducing BAX. In this study, we investigated the growth and adhesion of myeloma cells on hydrogel and collagen gels *in vitro* (see “Materials and Methods”). Based on the above data and signaling pathway map shown in the Supplementary Figure S2, we developed a signaling network module (integrin/FAK pathways) for dynamically simulating the effect of stiffness on adhesion and survival of MIC cells (Figure 2B).

### Increased expression of SDF-1 in the myeloma-associated bone marrow stromal cells

**Figure 3:**
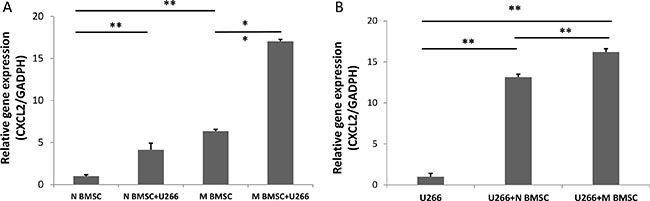
CXCL2 (SDF-1) expression in U266 and BMSCs (**A**) The expression level of CXCL12 in myeloma BMSCs was increased after coculture with U266 cells. N BMSC means healthy BMSCs, N BMSC+U266 means healthy BMSCs coculture with U266, M BMSC means myeloma patients’ BMSCs, M BMSC+U266 means M BMSC coculture with U266. (**B**)The expression of CXCL12 in U266 cells after coculture with healthy and myeloma BMSCs. U266 means U266 cells only, U266+N BMSC means U266 was collected after culturing with healthy BMSCs, U266+M BMSC means U266 was collected after culturing myeloma BMSCs. **p < 0.01.

In our previous studies, we found that SDF-1 regulated the stiffness of BMSCs *in vitro* to generate a better microenvironment for myeloma growth [22]. We compared the expression of SDF-1 in BMSCs collected from myeloma patients (myeloma BMSCs) and healthy volunteers (healthy BMSCs) with/without coculture of myeloma cells. As shown in Figure 3A, the SDF-1α mRNA level in the myeloma BMSCs was 6 times higher than that in the health BMSCs. The mRNA levels of SDF-1 were dramatically increased 4.13 ± 0.7 and 2.68 ± 0.2 times in both myeloma BMSCs and health BMSCs, respectively, when cocultured with U266 myeloma cells. We also compared the expression of SDF-1 in U266 myeloma cells. The expression of SDF-1 was significantly elevated about 16.2 ± 0.4 times and 13.1 ± 0.3 times when cocultured with myeloma BMSCs and healthy BMSCs, respectively (Figure 3B). Together, these results suggest that cell-cell interaction of myeloma cells and BMSCs enhance the expression of SDF-1 in both types of cells.

### Myeloma-BMSC interaction up-regulates TGFβ expression

**Figure 4:**
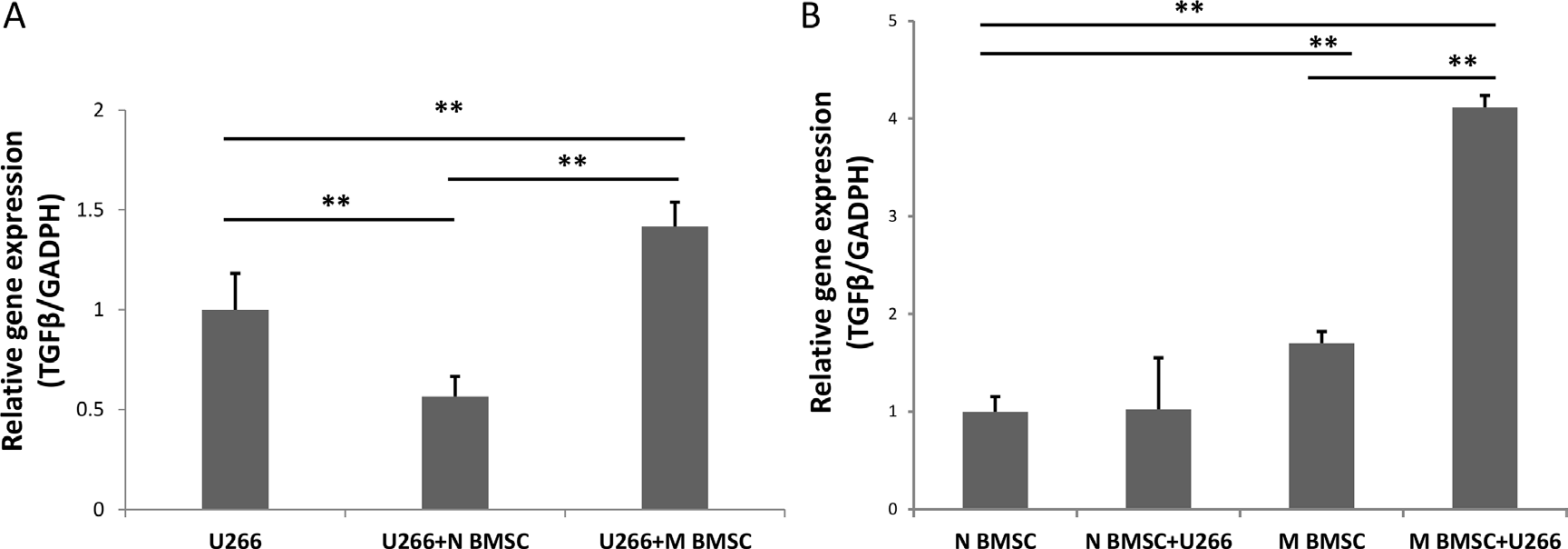
TGFβ expression in U266 and BMSCs (**A**)The expression of TGFβ in U266 cells after coculture with healthy and myeloma BMSCs. U266 means U266 cells only, U266+N BMSC means U266 was collected after culturing with healthy BMSCs, U266+M BMSC means U266 was collected after culturing myeloma BMSCs. (**B**) The expression level of TGFβ in myeloma BMSCs was increased after coculture with U266 cells. N BMSC means healthy BMSCs, N BMSC+ U266 means healthy BMSCs coculture with U266, M BMSC means myeloma patients’ BMSCs, M BMSC+U266 means M BMSC coculture with U266. **p < 0.01.

TGFß1 is a vital factor in regulating the balance of Tregs and CD8^+^ T cells; and increased expression of TGFß1 contributes to immune suppression in the MM microenvironment [3]. Here, we measured the expression of TGFß1 in the myeloma cells treated with or without BMSCs. Since both BMSCs and myeloma cells synthesize TGFβ [14, 23, 24], the expression of TGFß1 was determined in BMSCs and myeloma cells separately. There were dramatic changes of TGFß1 expression in U266 myeloma cells after cocultured with either healthy BMSC or myeloma BMSCs as shown in the Figure 4. A reduced expression of TGFß1 was observed in the U266 cells cocultured with healthy BMSCs (Figure 4A). In contrast, the expression of TGFß1 in the U266 cells cocultured with myeloma BMSCs was enhanced 1.4 fold. No significant changes in the TGFß1 expression were seen in the healthy BMSCs cultured with or without U266 cells (Figure 4B). In the myeloma BMSCs cocultured with U266 cells, the expression of TGFß1 increased about 2.4 times. Additionally, the basal expression level of TGFß1 in the myeloma BMSCs was about 1.7 times higher than that in the health BMSCs. Our results indicate that the interaction of myeloma with BMSC enhances TGFß1 expression in both cells.

### Model development

In this study, we established a hybrid multi-scale agent-based model (HABM) to simulate the development of myeloma in various bone marrow microenvironments in a three-dimensional space, and validated the performance of this model with experimental data. We hypothesize that 1) SDF-1 boosts MIC growth and protects MICs from chemotherapy-induced injury; and further drives the lineage process of multiple myeloma [11]; 2) myeloma-BMSC interaction induced secretion of TGFβ, which suppress the immune response during myeloma development. As shown in the Figure 1, myeloma development was simulated at intracellular, intercellular, and tissue levels. The HABM model described a simulated system including five types of cells, which were represented by five types of compartments of intracellular signaling events and interfaces. Supplementary Table S4 represents the parameters of ABM model, which were directly collected from some related studies or indirectly inferred based on the data produced in our laboratory. The above five compartments are shown below.

**Figure 5:**
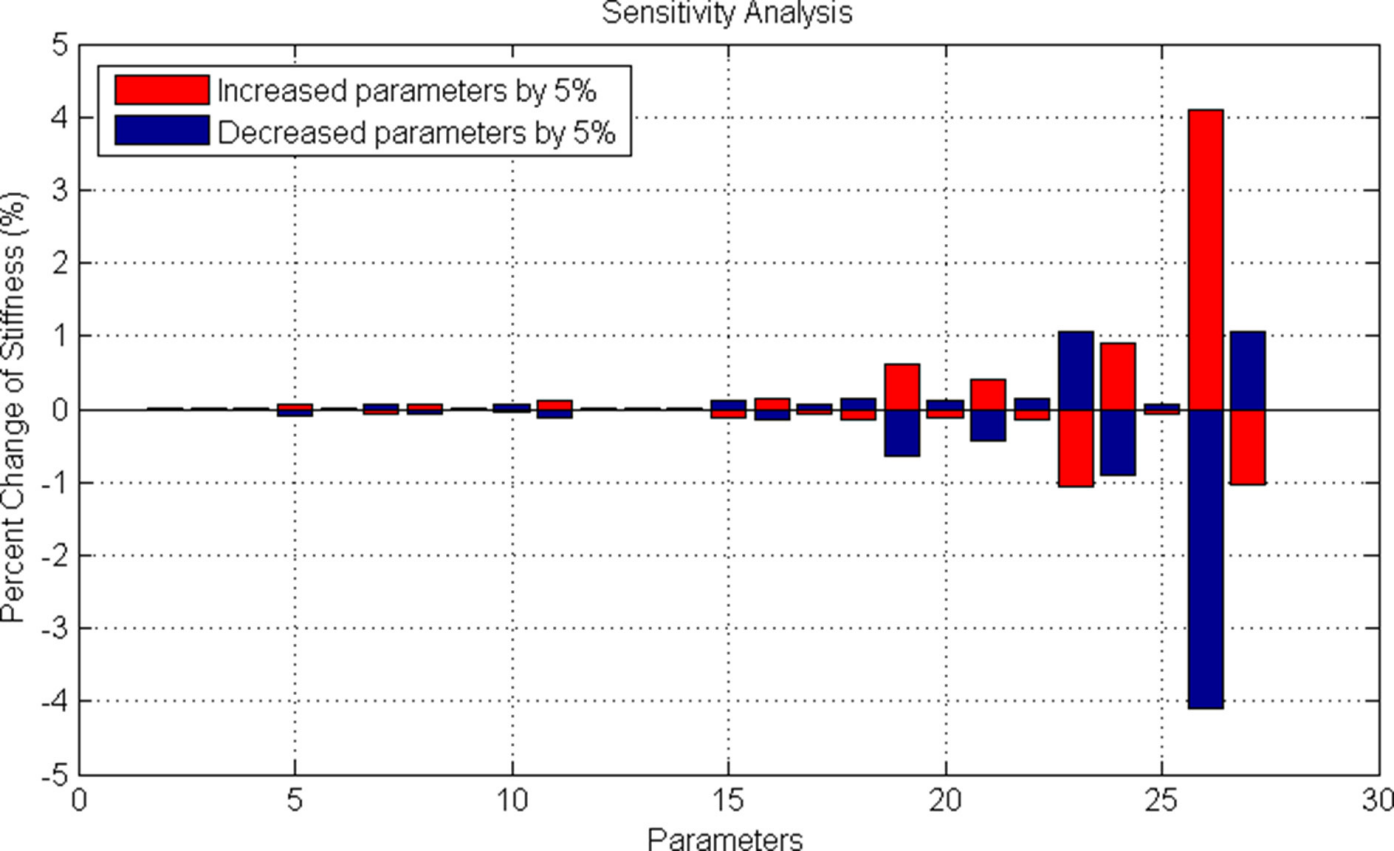
Sensitivity analysis of stiffness-related signaling pathways in BMSC cells

**Figure 6:**
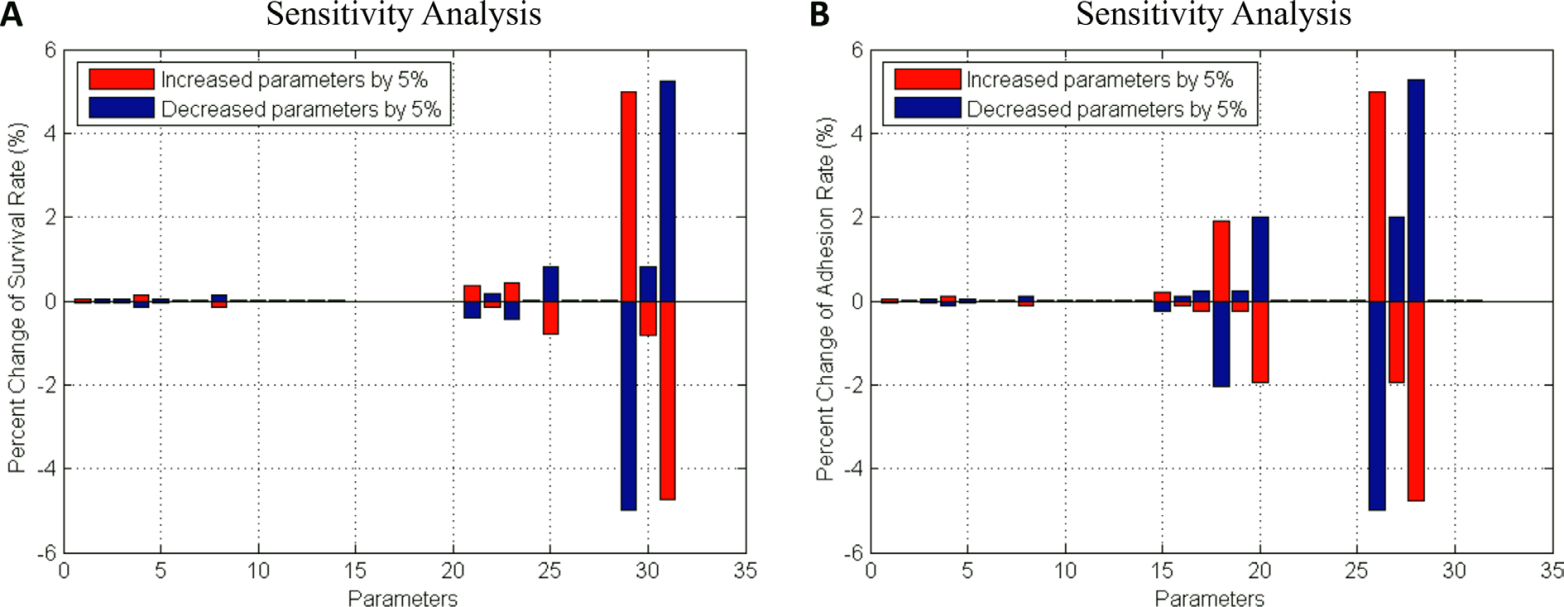
Sensitivity analysis of survival- and adhesion- associated signaling pathways in MIC cells (A) Survival rate (B) Adhesion rate.

(1) Bone Marrow Stromal Cell (BMSC) compartment. This compartment modeled the 3D reticular network formed by BMSCs. The changed stiffness of BMSCs in response to SDF-1 altered the three-dimensional distribution of bone marrow. Based on the signaling pathways shown in the Figure 2A, we developed an ODE system to simulate SDF1-triggered signaling transduction and BMSC responses. The details of this ODE system were described in the section “Materials and Methods”. All the parameters involved in this system were estimated using GA algorithm [25], and presented in the Supplementary Table S2. The predicted stiffness of myeloma BMSC via this ODE system was changed from 400 to 526pa following treatment with 5nM SDF-1, which was very close to the experimental results (400–530 pa) shown in the Choi's work [10]. To understand the relationship between ODE system outputs and variations in individual model parameter values, local parameter sensitivity analysis was performed [25]. The sensitivity analysis for these parameters indicated that this system was very stable (Figure 5).

(2) Myeloma Initiating Cell (MIC) compartment. This compartment represented the myeloma stem cells. MIC cells sensed the changes of stiffness from its local BMSCs and accordingly modulated their proliferation, differentiation, apoptosis, migration and adhesion. MICs can continuously generate MMs during the process of proliferation and differentiation [49]. Particularly, the adhesion and survival rate of MICs were simulated using an ODE system basing on the signaling pathways shown in the Figure 2B. The pathway map involved in this ODE system was determined according to the data shown in the Supplementary Figure S2 and others from the related literatures. All of the parameters in this system were also estimated using GA algorithm [25] and shown in the Supplementary Table S3. The sensitivity analysis of all the parameters demonstrated that this system was also stable (Figure 6).

The predicted outcomes of the ODE system for the intracellular signaling in MICs had a close similarity to the experimental data (Supplementary Figures S3–S4). The measured (Supplementary Figure S3A/S3B) and predicted (Supplementary Figure S3C/S3D) survival rates of MIC population with absence/ presence of BTZ under different collagen Gels (or hydrogels) were consistent. According to the analysis from Feng *et al*., the effect of collagen can be roughly equivalent to hydrogel: the stiffness on collagen 0.5 mg/ml and 0.75mg/ml are close to 200pa and 400pa, respectively [26]. Another aspect, the adhesion rate of MICs in the presence of BMSCs from myeloma patients is 67% (Supplementary Figure S4A), when compared the cells cultured with normal BMSCs, in which stiffness made great contribution on cell adhesion as 47% (Supplementary Figure S4B). Here, we assumed that other factors except stiffness totally have the same contribution (~20%) to different conditions. Therefore, the predicted adhesion rates of MICs from 100 pa to 400 pa were also close to the observations (Supplementary Figure S4C).

(3) Mature Myeloma cell (MM) compartment. This compartment represented the mature myeloma cell population which was differentiated from MICs.

(4) CD8^+^ (CD8^+^ CTL) T cell compartment. This compartment represented the Cytotoxic T Lymphocyte (CTL) population, which mediates lysis of myeloma cells through cell-cell contact. The activation and proliferation of CD8^+^ T cells reflected the immune response against the myeloma development.

(5) Regulatory T cell (Treg) compartment. This compartment represented the immune-suppressive T cell population, which is responsible for the maintenance of peripheral tolerance, and has been implicated in the suppression of tumor immunity. Tregs have been shown to inhibit tumor-specific T cell functions, such as the cytotoxic effects of CD8^+^ T cells.

The intercellular communications in HABM reflects the relationship between cancer cells, immune cells and tumor microenvironment evolution during multiple myeloma progression through the following five aspects. 1). The MIC-BMSC positive feedback loop is composed of SDF-1-triggered alteration in the biomechanical property of BMSC and BMSC-boosted proliferation of MICs [11]. MICs secreted SDF-1 into their surrounding extracellular matrix [28]. SDF-1 is then diffused in the 3D ECM. Responding to the stimulation from the local SDF-1, the stiffness of BMSCs is altered via activated SDF-1/CXCR4 signaling pathway [10]. MICs sensed those changes and attached on the stiffer BMSCs in the local position [26]. Once attached, the growth factors secreted by BMSCs boost the survival, proliferation, and differentiation of MICs, and thus drive multiple myeloma growth. The BMSCs also protected MICs from the treatment of drugs, such as BTZ [29]. 2). The lineage process of myeloma expansion is illustrated by the dynamics of two types of myeloma proliferation. Myeloma cells (MM) are generated not only from MIC differentiation but also expansion by themselves [11]. 3). CD8^+^ T cells recognized MICs and MMs in their local regions, and migrate toward these target cells for clearance [3]. 4). Accumulation of Treg cells potentially inhibit the generation of CD8^+^ T cells, resulting the suppression of CTL-mediated antitumor immune responses [27]. 5). The secretion of TGFβ from both myeloma and BMSCs lead to an increased proliferation of Tregs and inhibited proliferation of CD8^+^ T cells, resulting a suppression of immune response [14]. The myeloma-BMSC interaction impair the CTL-mediated lysis of multiple myeloma cells via the increased production of TGFβ [30].

The tissue scale of our HABM model reflects the 3D cancer growth via various intercellular cell-cell interactions spatially and temporally. These interactions include SDF-1-induced MIC-BMSC positive feedback loops, the myeloma lineage process from MIC to MM, TGFβ-induced immune suppression, and CTL-mediated target cell lysis etc. At the tissue level, intracellular signaling pathways are triggered by the local SDF-1 or TGFβ via the interfaces of cell agents, and the changes in the cells’ fate and behaviors in turn modulate the environment for cell growth. In this scale, the dynamic 3D distribution of SDF-1 is defined by the secretion of SDF-1 from MICs and the diffusion of SDF-1 in the 3D ECM. TGFβ can be secreted from both BMSC and MM, and the dynamic 3D distribution of TGFβ is defined by the diffusion of TGFβ in the ECM. TGFβ plays an important role in regulating the immune system. Chemical microenvironment is determined by the drug concentration in the bone marrow. In addition, the tissue stiffness defined by BMSC contraction determined the biophysical microenvironment. The cell behaviors, such as proliferation and migration in 3D ECM, determined the distribution of cell populations in bone marrow.

Multiple myeloma cells at different stages of differentiation were initially in the bone marrow microenvironment. We used 100, 100, 20 and 5 cells of MIC, MM, CD8^+^ T cells, and Tregs, respectively, to mimic the initial stage for myeloma spreading to a new location in the bone marrow. The ratio of Treg/CD8^+^ was determined based on the previous findings [31]. In addition, BTZ, LEN, and Thal were used as the representatives of cytotoxic and immunomodulatory drugs in this model to test the response of myeloma cells and immune cells. These drugs were applied alone or combined with various doses. The treatment duration was scheduled with 144 hours (6 days) in HABM, which represented the acute drug effects in clinic. In HABM model, a set of parameters for the dosage regimen of three drugs indicates a treatment condition. Once the variables related to drug dosages were assigned with certain values, the cells response to the “treatment” by changing their apoptosis and proliferation rates. The number of each cell population in HABM was counted every 2 hours. The drug effects were quantitatively identified from the changes in living tumor cell and immune cell populations following treatment. In this model, we simulated the tumor and immune responses up to 600 hours.

Moreover, we defined synergy effect index of three-drug combination, and quantitatively examined the synergism among these three drugs. Eleven doses of each drug and their combination with other drugs were then examined in the established HABM model; and each treatment condition simulated for 200 times. Time resolution was 2 hours. Totally 1331 conditions were simulated for 266,200 times using the parallel computation on TACC (http://www.tacc.utexas.edu) with 1400 cores.

This is the first time to apply mathematic model for simulating tumor growth and immune response within an integral system. Supplementary Figure S5 shows simulation examples for myeloma growth in the bone marrow microenvironment and the effects of immune system on myeloma cell growth. Supplementary Figure S5Ai–S5Aiii represent the dynamic changes of myeloma cell growth in the absence of immune system in the bone marrow microenvironment. Supplementary Figure S5Ai indicates that the growing rate of MM is obvious faster than MIC, consistent with the experimental results reported by Yang. *et al*. [32]. The simulation results for myeloma cell growth at 100 hour and 600 hour were shown in the Supplementary Figure S5Aii–Aiii, respectively. When the immune cells were added in this system, the simulated growth for MICs and MMs obviously slowed down (Supplementary Figure S5Bi). Supplementary Figure S5Bii–Biii show that myeloma cell growth at 100 and 600 hour in the presence of the immune cells. The results from the Supplementary Figure S5 indicate a negative regulatory role of the immune system to myeloma cell growth. Supplementary Figure S5Bii–Biii represent multiple myeloma cell killing by CD8^+^ CTL, however, the anti-MM effects was interfered by MM-induced immune paresis. The model shown in Supplementary Figure S5B was further perturbed with two types of combination treatment strategies, and the dynamic changes of each cell compartment are presented in the following sections.

### Model validation

**Figure 7:**
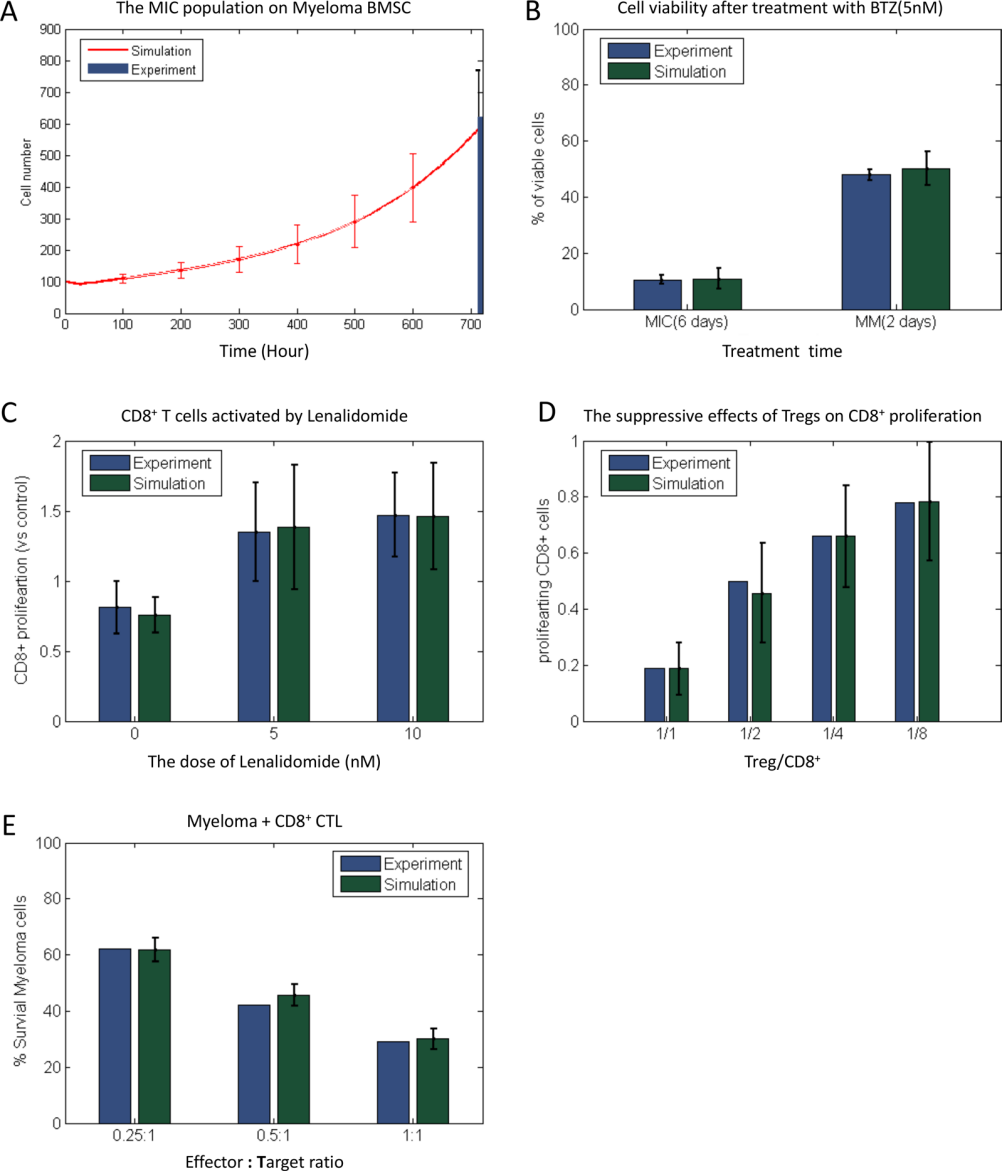
Comparison of simulation results and experiment observation The simulation results of MIC populations (**A**) myeloma cell viability after treatment with BTZ (**B**) LEN-induced CD8^+^ T cell proliferation (**C**) the suppressive effects of Tregs on CD8^+^ proliferation, and CTL-induced lysis of myeloma (**E**) under Myeloma-BMSC were compared with published experiment results.

The simulation results from our HABM model were validated using experimental results from our laboratory and previously reported studies. We firstly modeled MIC progression in the microenvironment without the immune system and compared our results with the ABM model reported by *Su, et al*. [11]. When the immune system was absent, the simulated MIC populations in a microenvironment with BMSCs was expanded 5.98 times after 4 weeks (Figure 7A), which is consistent with the experimental observations reported in Feng's work [9], and is also close to the predicted results in *Su, et al’s* model [11]. However, the HABM model reflects more pathogenesis-associated factors. For example, (1) HABM is able to demonstrate the significant difference of myeloma development in the microenvironment with/without the immune cells (Supplementary Figure S5); and (2) tumor growth is much slower in the microenvironment with anti-tumor immune response (Supplementary Figure S5). Thus, the computational models with the immune system will lead to a more accurate prediction of tumor development and drug treatment effects.

In the absence of the immune cells, we then predicted that the response of myeloma cells treated by 5 nM BTZ was close to the experimental outcomes (Figure 7B). The predicted cell viability of MIC cells on the day 6 after treatment was reduced 89.2%, which was close to our experimental result shown in the Supplementary Figure S3. The number of myeloma cells was reduced 49.5% following two days treatment, which was also consistent with Campanella's work [2]. We further analyzed the effects of LEN on the proliferation of CD8^+^ T cells (Figure 7C). The treatment with 5 μM and 10 μM of LEN led to 38.4% and 46.4% increase in the number of CD8^+^ T cells, respectively. These results were also consistent with the data reported previously [33]. Moreover, we predicted the suppressive effects of Tregs on CD8^+^ T cells proliferation when combined with different ratio of Treg to CD8^+^ T cells. As shown in the Figure 7D, the simulated results were similar to those from Fostier's work [34]. Finally, we predicted the CD8^+^ T cells-mediated lysis of myeloma cells (Figure 7E). The survival rate of myeloma cells (MIC and MM together) in presence with three different ratios of CD8^+^ T cells were close to the experimental results from Haart. *et al* [3]. These above simulation results suggest that our HABM model is of high accuracy.

### Effects of therapeutic drugs on myeloma development

**Figure 8:**
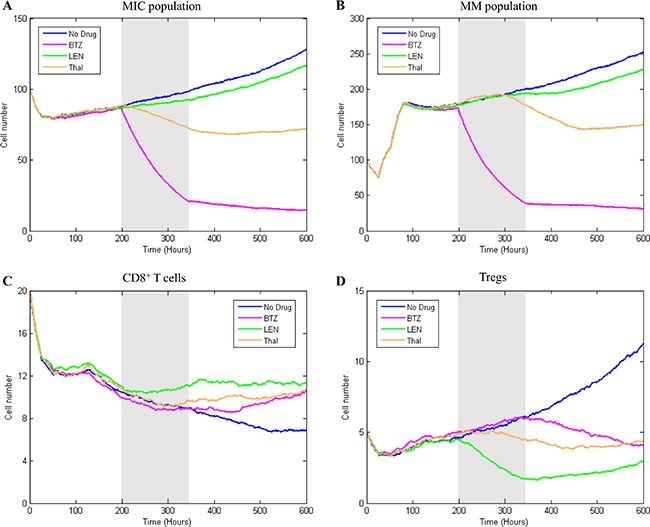
The treatment effects of single drug on four types of cells The simulation results of single drug treatment effect on MIC (**A**) MM (**B**) CD8^+^ T cells (**C**) and Tregs (**D**) were compared with three strategies.

To explore the resistance of myeloma cells to chemo- and immuno- therapeutic drugs and the risk of tumor relapse in a simulated bone marrow microenvironment, we modeled the outcomes from the treatments with BTZ, LEN, and Thal, respectively. Multiple myeloma was sensitive to BTZ, so that anti-cancer therapy using BTZ induces direct tumor cell apoptosis by activating Caspase-8/9 mediated apoptotic pathways [14, 35]. According to Campanella's experimental results, the dose 1.5 nM of BTZ was a turning point to the myeloma cell killing [2]. Therefore, we used 1.5 nM BTZ in our model for this simulation. Different from BTZ, the most important working mechanisms of IMiDs (such as, LEN and Thal) include T-cell co-stimulatory, suppression of Tregs, and disruption of myeloma cell -BMSC interaction [14]; Thus, a relative high dose (10 μm) was used for both LEN and Thal in our model [1, 33]. Figure 8 represents the effects of all three drugs on each type of cells. The growing rates for the untreated MIC and MM were obviously increased following time; Once BTZ was delivered to bone marrow, the populations of MIC and MM were sharply killed (Figure 8A–8B). As a widely used immunomodulatory drug for the treatment of multiple myeloma, LEN re-activates the immune system in bone marrow, resulting in suppression of Treg cells and activation of CD8^+^ T cells [12, 36]. Compared with BTZ and Thal, LEN effectively increased the proliferation of CD8^+^ T cells and inhibited the proliferation and function of Tregs (Figure 8C–8D). Considering the special inhibiting effects to SDF-1/CXCR4 signaling pathway in MM [1], Thal indirectly inhibits the proliferation of myeloma cells by regulating the SDF-1-trigged stiffness of BMSC and further decreases the secretion of TGFβ from BMSC. Figure 8A–8B show that the treatment with Thal induced suppressive effects on both MIC and MM cells. Together, these simulating results suggest that: (1) BTZ alone resulted a significant killing in the cell numbers of MIC and MM; (2) LEN or Thal alone didn't lead to a significantly kill of the tumor cells but to recover the immune system.

### Combined drug effects on disease development

**Figure 9:**
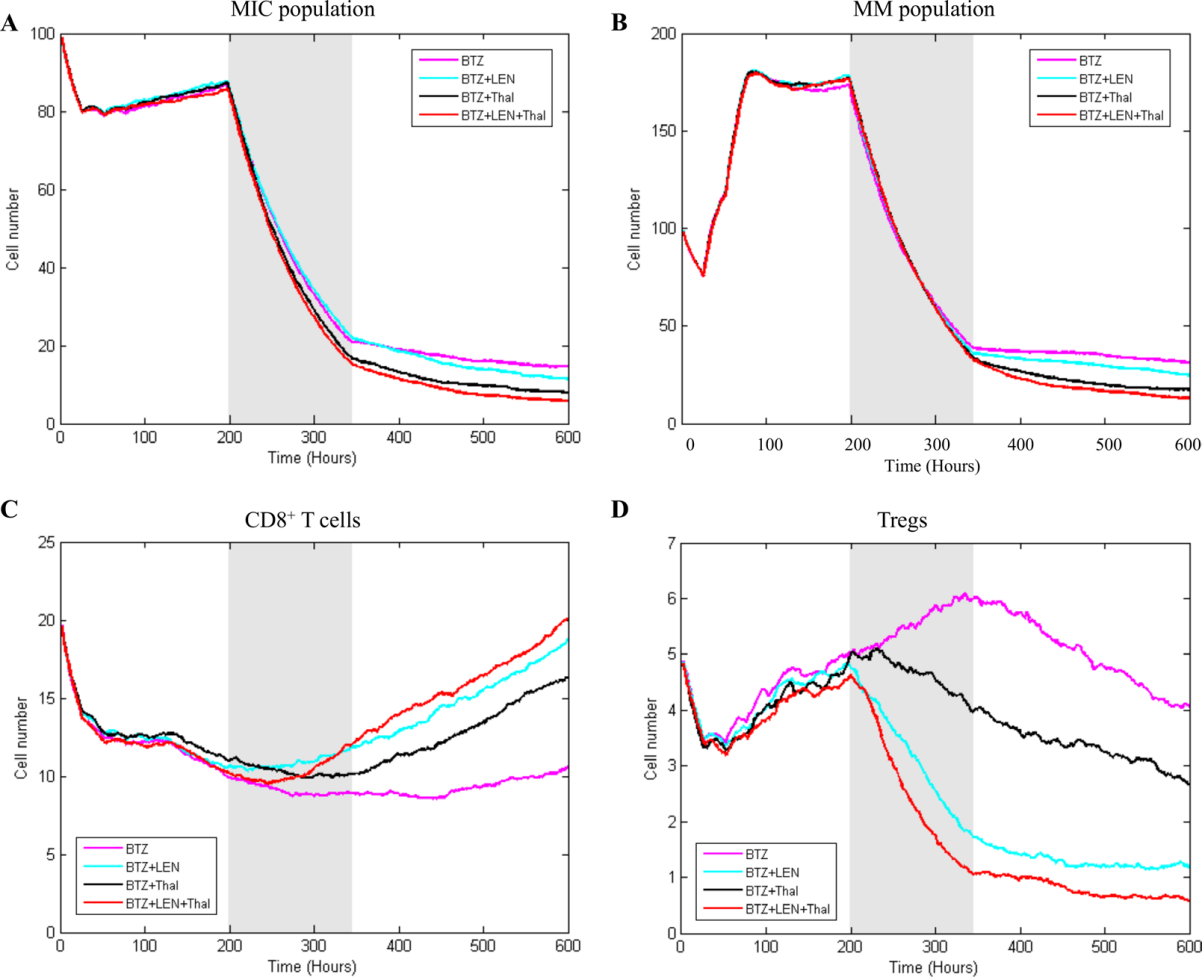
The effects of combined treatment on four types of cells The simulation results of multiple drug treatment effect on MIC (**A**) MM (**B**) CD8^+^ T cells (**C**) and Tregs (**D**) were compared with three strategies.

As shown in Figure 8, treatment multiple myeloma with individual drug didn't obtain desired effects [35]. In this section, we simulated the combined drug effects on myeloma development to investigate optimal therapeutic strategies. Our rationale is that an optimal therapeutic strategy should consider multiple potential targets in bone marrow for complete clinical responses. Here, we focused on the BTZ-based combined effects, in which chemotherapy and immunotherapy drugs were delivered to multiple myeloma in bone marrow, simultaneously. Figure 9 shows three strategies of drug-combinations and the corresponding effects on myeloma growth and immune response simulated by our model. Treatment with multi-drug presents obvious stronger effect than that with a single drug. This is consistent with the Wang's work [37]. The treatment efficacy on multiple myeloma with BTZ plus Thal is better than that with BTZ plus LEN (Figure 9A–9B). Triple combination with BTZ, LEN, and Thal results in the lowest survival rate of both MIC and MM cells (Figure 9A–9B). The enhanced anti-tumor effect appears to be due to the increased CD8^+^ T cells activity. Combined treatment with BTZ, LEN, and Thal maximizes leads to a quick increase in the population of CD8^+^ T cells (Figure 9C), and a suppression in the proliferation and function of Tregs (Figure 9D). Together, these findings indicate that the combined therapy with three drugs (BTZ, LEN, and Thal) results in an excellent treatment response, which is due to a direct antitumor effect and enhances the host's antitumor immune responses, simultaneously.

### Drug synergy evaluation

We tested the combined effects of three representative drugs, BTZ, LEN, and Thal, to examine if induction of direct tumor cell apoptosis, disruption of the MIC-BMSC interactions and enhancement of anti-tumor immune response have clinical potentials for multiple myeloma treatment. For each drug, 11 doses (0 as no drug, 1–10 levels from 0.1 × to 1 × to the original dose) were selected and the full combinations of these three drugs were explored for drug efficacies. Each combinatorial condition was simulated for 200 times at 300 time points (600 hours) for 5 cell types, and totally 266,200 data sets were generated. For each combined strategy, the means of 200 replicates were used for further analysis.

Here, we defined synergy effect index (*C_i,j,k_*) of three-drug combination as following:

$$C_{i,j,k}=\frac{D_{i}D_{jk}+D_{j}D_{ik}+D_{k}D_{ij}-2D_{i}D_{j}D_{k}}{D_{i,j,k}}$$ (1)

Where *i, j*, and *k* are the doses for BTZ, LEN, and Thal, respectively, and *i*, *j*, i, j, k∈{1,…,10} *D* denotes the survival ratio of myeloma cells after treatment by drugs. *D_i_*, *D_j_*, and *D_k_*,represent the cell viability after treated with single drug; and *D_ik_*, *D_ij_,* and *D_jk_* with two drugs. In formula (1), the numerator and denominator indicates the expected response effects [38] and simulated effects of triple-combination, respectively. The effect is synergistic if the value of *C_i, j, k_*_> 1_, antagonistic if *C_i, j, k_*_< 1_, and additive if *C_i, j, k_*_= 1_. We presented four cases about the synergy effects of three-drug combination (Supplementary Figure S6). For example, we randomly set Thal with dose level 10, the synergy has been obviously found between BTZ (level 1 to 7) and LEN (level 1 to 10) in Supplementary Figure S6A; It also consistent with some previous works that LEN potentiates the activity of BTZ in preclinical studies [35, 39, 40]. If the dose of BTZ is low, the synergy effects occurred when the doses of Thal and LEN are increased (Supplementary Figure S6–S6D). When the dose of LEN is low (immune system was not activated), the synergy effects was induced when Thal is increased (Supplementary Figure S6C).

## DISCUSSION

This work focused on studying the effects of cell-cell interaction on myeloma development in bone marrow microenvironment, and cast new light on the strategies to overcome immune suppression and improve the relapse of multiple myeloma. The *central hypothesis* of this work is that myeloma development in the bone marrow is promoted by: (1) the positive feedback loop between MICs and BMSCs via SDF-1 and the increased stiffness in the BMSCs niche; (2) cell adhesion-mediated immune resistance against CTL function induced by TGFβ. These are the main reasons responsible for immune tolerance [3], drug resistance and cancer relapse [11], and have been successfully covered in our hybrid multi-scale agent-based model.

We are the first to study systemic modeling of tumor growth, drug response, and immune response within an integrated 3D system (Figure 1). In the above section, we briefly introduced one of our previously reported works (Su, *et al*.) using an ABM model to simulate the effects of SDF-1-induced chemo-physical communications among MICs and BMSCs on myeloma lineage process [11]. We also used the well-defined SDF-1-induced chemo-physical communications between MICs and BMSCs in our HABM model and simplified the lineage process MICPCMMTMM by using MICMM [49]. For the model structure, we borrowed the idea of hybrid model from Solovyev's work, which combines ODEs and ABM in a single computational system [43].

Our contributions are summarized as below:

Firstly, *Su, et al*. didn't take the effects of immune system into consideration, which plays a key role in tumor progression and drug resistance in human. Our work is the first one to study systemic modeling of tumor growth and immune response within an integrated 3D system. We creatively added two components “CD8^+^ T Cell” and “Treg” in our model to represent a basic immune system with key functions, including CTL-mediated target cell lysis, and Treg-induced suppression of CTL proliferation.

Secondly, comparing with the two-dimensional hybrid model developed by *Solovyev et al*., our 3D hybrid multi-scale model mimics signal transduction processes at the intracellular scale, stochastic cell behavior at the intercellular scale, and the diffusion of growth factors and drugs within the microenvironment at the tissue scale.

Thirdly, we defined the synergy index of the three-drug combinations and predicted the combined effects of three drugs with multiple doses in an ABM system. While most of previous models only studied the synergy effects between two drugs in the ABM models or signaling pathway networks [45–48].

In our HABM model, an ODE system modeled the SDF-1-triggered signal transduction process to determine the changes in the biophysical property (stiffness) of BMSCs (Figure 2A and Supplementary Figure S7). The increased stiffness in BMSCs promoted the proliferation of MICs, which was also processed by another ODE system (Figure 2B and Supplementary Figure S4). In the result section, we introduced the local sensitivity analyses of parameters in ODEs by increasing or decreasing one parameter at a time. Moreover, we discussed the uncertainty analysis [44] for all the parameters.

### Uncertainty analysis

Based on the estimated parameters shown in the Supplementary Tables S2–S3, we obtained two baseline ODE models. We then generated the testing samples, in which all of the parameters were increased or decreased randomly with uniform probability within a given range (5%). Each testing sample denoted a set of new parameters, which was then used to execute the related ODE model. Considering the search space of testing samples is quite large, we randomly generated nine statistical experiments with different sample sizes, and evaluated the variability of model results with each sample set. The numbers of testing samples in nine experiments were significantly different (e.g. 30, 100, 200, 500, 1000, 2000, 5000, 8000, and 10000). Supplementary Figure S8 shows the variability of stiffness for all testing cases in each experiment. The mean and standard deviation were normalized by the output from the baseline model (the green line in Supplementary Figure S8). When the size of a sample set is larger than 5000, the mean values of outputs converge to the steady state and the changes in outputs would be less than 4% eventually. Supplementary Figure S9 shows the variability of survival rate and adhesion rate for all testing cases in each experiment. Similarity, when the sample size is larger than 5000, the mean values converge to the steady state and the percentage changes of outputs in all cases eventually would be less than 5%. Our proteomic data was represented as fold changes comparing with the time point 0 (Tables S1–S2 in Supplementary Data File), thus, we executed all the testing cases on each ODE model with the same initial state of the related network (each protein node was assiggend as “1”). In summary, Supplementary Figures S8–S9 indicate that the constructed pathway models in BMSC and MIC cell agents are stable.

We also provide a novel computational platform for evaluation of the cellular responses to the single and combined drug treatment. Wang. *et al*. performed a meta-analysis for the efficacy and safety of combined treatments (BTZ plus LEN/Thal vs BTZ or LEN/Thal) containing regimens as the induction therapies in newly diagnosed multiple myeloma [37]. Their analysis suggests that BTZ plus LEN or BTZ plus Thal resulted a significant increase in the clinical responses of patients compared with those received a single drug. Our simulated results also indicated that BTZ-based combined treatments were more efficient than the single drug strategy (Figures 8, 9). In addition, we found that predicted survival outcomes from the combined therapy with three drugs were greatly improved (Figure 9), consistent with the results from clinic indicating that the use of BTZ, LEN and Thal dramatically changed outcomes for patients with relapsed myeloma [41]. Our simulations indicate that the combined therapy with three drugs (BTZ, LEN, and Thal) results in an excellent treatment response rather than other combinations. In addition, the maximal dose of BTZ, LEN, and Thal simulated in our model were 5 nM [2], 10 μM [33], and 10 μM [1], respectively. Clearly, this model facilitates us to identify the optimal dose setting of combination therapeutic options for improving survival outcomes.

Although the parameters of the HABM model were tuned manually or determined from some related literatures, the proposed model under different contexts is capable to re-capture the experimental observations with high precision.

(1) Pro-oncogenetic myeloma-associated BMSC microenvironment. Without the intervention of immune system, the MICs population was boosted in myeloma-associated bone marrow and thus driven the development of myeloma (Figure 7A).

(2) The response of myeloma cell to BTZ treatment. The predicted dose effects of BTZ on cell survival (Figure 7B) was consistent with the experimental results (Supplementary Figure S3 and [2]), indicating that our model has the capability to predict the drug responses of myeloma.

(3) The efficacy of LEN in activating CD8^+^ T cells. When the representative IMiD drug LEN was delivered with a wide range of doses, CD8^+^ T cells were efficiently activated and the proliferation was promoted obviously. The predicted outcomes and the experimental observations were very close (Figure 7C).

(4) The suppressive effects of Tregs on CD8^+^ proliferation. Figure 7D represents that the suppressive effects of Tregs on CD8^+^ T cell proliferation without any drug perturbation [34].

(5) CD8^+^ T cells mediated lysis of myeloma cells. Myeloma cells were significantly lysed by the cytotoxic effect of CTL when CD8^+^ T cell population was expanded [3]. Our predicted results were consistent with these observations (Figure 7E).

In summary, we proposed a 3D hybrid multi-scale agent-based model (HABM) to reveal the molecular mechanisms associated with cancer development and immune response in an integrated tumor microenvironment. In the HABM system, not only cell-cell interactions were modeled for describing various types of intercellular communications, but also the key signaling pathway networks were stimulated for elaborating the intracellular signal transduction processes. Under different perturbed conditions, the predicted outcomes of HABM model were very close to the experimental observations, which proved that the simulation results of our model are reliable. This is the first time to model the cancer development in a complicated system with immune component, so that the stimulated microenvironment was more close to the condition *in vivo* rather than previous works. This study also provided a novel computational tool to quickly predict the drug treatment response of multiple myeloma, which is good for us to find the best treatment strategy of multi-drug and the optimal dose combination.

## MATERIALS AND METHODS

### Experiment

### Cells and culture

### Myeloma cells

Human myeloma cell line RPMI8226 was obtained from the American Type Culture Collection. RPMI 8226 cells were cultured in RPMI 1640 (Hyclone) containing 10% heat-inactivated fetal bovine serum (Sigma), 2 mmol/L l-glutamine (Invitrogen), 100 U/mL penicillin and 100 μg/mL streptomycin (Invitrogen).

### Bone marrow stromal cells (BMSCs)

Myeloma BMSCs were isolated from remaining bone marrow samples of myeloma patients for routine diagnostic. Cells in the bone marrow sample were obtained by Ficoll density gradient centrifugation (AXIS-SHIELD). The cells were then plated in the tissue culture flasks at a concentration of 10^6^ cells/mL in Mesencult basal medium supplemented with MSC stimulatory supplements (both from Life technology). After 24 h incubation at 37°C in a 5% CO^2^ humidified atmosphere, non-adherent cells were removed, and the adherent fraction was cultured in fresh medium. Cells used for future experiment were no more than 10 passages. Normal BMSCs cell line HS5 was obtained from ATCC, and cultured in Mesencult basal medium supplemented with MSC stimulatory supplements.

### Coculture of myeloma cells and BMSCs

For coculture experiments, myeloma cells were seeded onto BMSCs pre-cultured on 6-well plates. Cells were cultured in RPMI 1640 with 10% FBS at 37°C in a humidified atmosphere of 21%O_2_ normal or 5% O_2_ hypoxic condition. Suspended myeloma cells were collected by repeated gently rinsing mixture. BMSCs were scraped for following RNA extraction.

### Reverse transcription-quantitative polymerase chain reaction (RT-qPCR)

Total RNA was extracted from the collected myeloma cells using Qiagen mini RNA extraction kit (Qiagen), according to the manufacturer's instructions. A total of 1 μg RNA was reverse-transcribed into cDNA using a SuperScriptIII first-strand synthesis system (Life Technology). RT-qPCR was performed using an Applied Biosystems Fast 7500, with endogenous GADPH used as a reference. The gene expression levels of transforming growth factor (TGFβ) and CXCL12 (SDF-1) were determined in each group. The primers used in this study designed and synthesized by Integrated DNA technologies. Primers were as follows: GADPH, 5′-3′GAGTCAACGGATTTGGTCGT (forward) and 5′-3′ TTGATTTTGGAGGGATCTCG (reverse); TGFβ, 5′-3′ CGTGGAGCTGTACCAGAAATAC (forward) and 5′-3′ CACAACTCCGGTGACATCAA (reverse); and CXCL12, 5′-3′ CAGAGCCAACGTCAAGCA (forward) and 5′-3′ AGGTACTCTTGGATCCAC (reverse). The cycle conditions were as follows: 1 min at 60°C, 10 min denaturation at 95°C followed by a total of 40 cycles 95°C for 15 sec and 60°C for 1 min.

### Proteomics data for SDF-1 signaling in myeloma BMSC

To model the SDF-1 signaling network of BMSC cells shown in Figure 2A, we collected phosphor-protein data from three sources. (1) The myeloma BMSCs were treated with SDF-1 (100 ng/ml) and the protein levels of *p*-FAK, *p*-MYL2, and *p*-RhoA were detected at 0, 5, and 10 minutes shown in the Choi's work [10]. (2) We did the similar experiment with SDF-1 (100 ng/ml) and also obtained the phosphorylation of *p*-FAK, *p*-MYL2, and *p*-RhoA at 0, 15, and 60 minutes [22]. (3) We did the RPPA analysis for the myeloma BMSCs treated with 100 nM/mL of SDF-1. This RPPA dataset covered four time points: 0, 5, 10, and 15 minutes. From this dataset, we collected the phosphorylated protein levels of ERK and MEK at above four time points. The phosphor-state of each protein was normalized against its expression at time point 0.

### Proteomics data of MIC

Similarly, we obtained the phosphorylation of key proteins for modeling the signaling network of MIC shown in Figure 2B. Side population of myeloma cells seeded on 100 Pa and 400 Pa surfaces and the protein samples collected at 0, 30, 60, and overnight for RPPA analysis. For each protein, the expression levels are normalized against its *t* = 0 min level. Finally, we collected the expressions of FAK, PI3K, AKT, JNK, c-Jun, and NFKB from this RPPA dataset because they were covered in the topology of network shown in Figure 2B. The details of the above proteomic data were described in Supplementary Data File.

### Cell population

### MIC growth and response to BTZ on the premade collagen gels with various stiffness

Two hundred of the side population (SP) of U226 cells were mixed with premade collagen gel with various concentration as shown in Supplementary Figure S3A. The Cells were allowed to grow in the gel for 1 week and then treated with or without 5 nM BTZ for six days. Cell viability was determined using MTT assay. The details of relative cell viabilities were represented in the Supplementary Figure S3B.

### Adhesion of MIC Cells on and the premade collagen gels

The Side population of U226 cells was seeded on the premade collagen gel with various stiffness. Twenty four hours after incubation, the attached cells were trypsinized and counted. The details were represented as Supplementary Figure S4A–4B.

### Agent-based model of myeloma development

We defined five types of agents in the ABM model to represent BMSC, MIC, MM, CD8^+^, and Treg, respectively (Figure 1). ABM a simplified model used for predict the effects of cell-cell interactions on growth and drug response of myeloma cells in a simulated bone marrow microenvironment. We initialized the bone marrow microenvironment as a cylinder 3D rectangular framework with evenly scattered BMSCs in the 3D extracellular matrix and central distribution of mixed MIC, MM, CD8^+^, and Treg compartments as a sphere. This multi-scale modeling includes intracellular, intercellular and tissue scales, which are illustrated in the Figure 1, and described into details in the following sections. Detailed flowcharts of each agent are illustrated in the Supplementary Information.

### Stochastic simulation of cell behaviors

**Figure 10:**
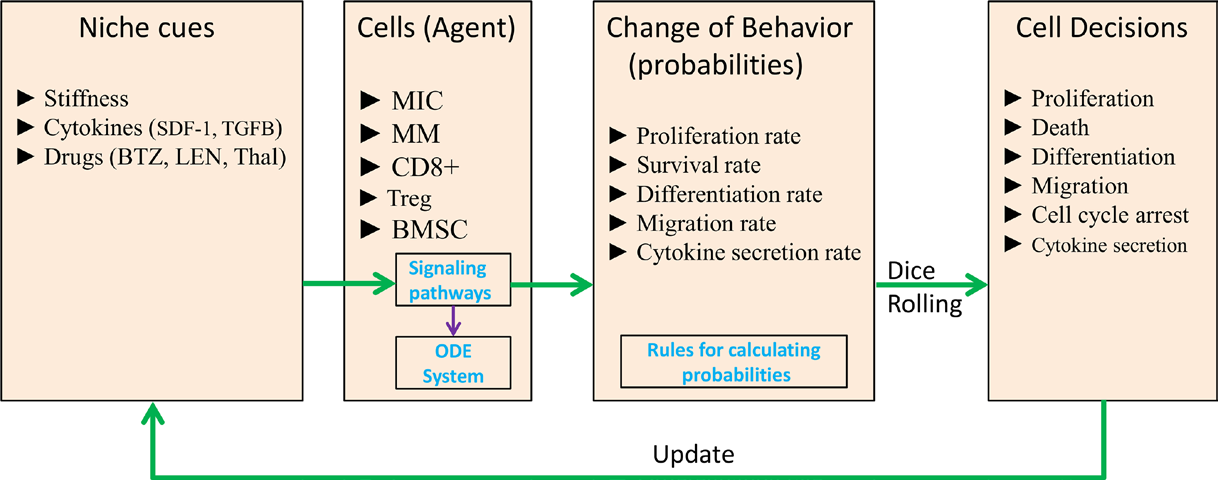
The stochastic simulation of cell behaviors

The Markov Chain Monte Carlo approach in the ABM model was used to simulate individual cell behaviors. As shown in the Figure 10, cell behaviors are simulated by probability-based rule implementation. A cell senses the hints in its neighborhood such as stiffness, local cytokines (SDF-1, or TGFβ) and drugs (Bortezomib, Lenalidomide, and Thalidomide) and adjusts itself with the imbedded signaling pathways, and outputs the corresponding changes in its cell behaviors, including proliferation, survival, differentiation, migration, and cytokine secretion rate. Cell decision is then determined by rolling a dice and compared with the probability threshold of a cell behavior. Details of each cell behavior for each agent as well as the corresponding rule are discussed in the following sections as well as the Supplementary Information.

### Intracellular scale

The biomechanical phenotype for each BMSC agent is determined using our developed ODE system to describe the effect of SDF-1/CXCR4 signaling pathway on local stiffness in response to the changes of in-situ SDF-1 concentration. SDF1-triggerred BMSC stiffness is described into details the following section.

The survival rate of MIC cells is determined by both the local ECM stiffness and the local doses of BTZ via a developed ODE system. Adhesion rate represents the probability of a MIC cell attaching on a BMSC agent with various stiffness. The ODE system for adhesion and survival of MIC cells has been described into details the following section.

The response of MM cells to local stiffness in terms of the possibilities of cells to enter the proliferation, apoptosis, and migration status are calculated using Hill functions. Similarly, Hill functions are also applied for denmondtration of the probabilities of proliferation, apoptosis, and migration for immune-related cell agents (CD8^+^ and Treg). Cell decision making process is defined by the agent rules with such probabilities as the major inputs.

### Cell fate determination

Once a MIC has decided to re-enter cell cycle, its microenvironment will be a key determinor for its fate. A MIC can either devide into two daughter cells, known as self-renewal, or differentiate into two MM cells. The probability of each fate is calculated using the Hill functions, and the decision making is realized by the die casting simulation.

### Intercellular scale

In response to the changes in the biomechanical properties of its microenvironment, a myeloma cell (MIC or MM) will proliferate, migrate, become quiescent, or undergo death process. We described those responses at the intercellular scale. In addition, a myeloma cell might be killed by CD8^+^ T cell in the neighborhood. Furthermore, when the Treg cells migrate towards CD8^+^ T cells, the proliferation of CD8^+^ T cells will be suppressed, due to cell cycle arrest or apoptosis of these cells.

### Migration

A non-M-phase cell will migrate if it can find a free space nearby. In the BM, MIC cells tend to migrate to the surface of the BMSC cells and attach on it. Their proliferation will be promoted with the increased stiffness of BMSCs. CD8^+^ T cells are apt to move towards the places of where myeloma cells reside. Treg cells migrate to CD8^+^ T cells and affect the proliferation of these effector cells in a manner of cell cycle arrest or apoptosis. The migration was governed by space availability, migration speed, and stochastic effects using Hill functions and die-casting simulation.

### Division of MIC and MM agents

If M-phase cells are found at least at one free location within the searching distance, there cells will divide. It follows the same migration rules, but has a much smaller migration distance. Thus, the de novo daughter cells will always be next to the parental cells. If no space is available, the cells will remain in M-phase and obtain entrance in the next cycle.

### Lysis of MIC and MM

Once recognizing the location of myeloma cells (MIC and MM) in its neighborhood, the CD8^+^ T cell will migrate towards them. When a CD8^+^ cell is adjacent to a certain myeloma cell, the lysis will occur. The myeloma-BMSC interactions can promote the myeloma cells escape from the immune system, therefore, we also defined the corresponding rules to reflect a fact that CD8^+^ cells prefer to kill the target tumor cells which are not attaching on the BMSCs.

### Suppression of CD8^+^ T cell proliferation

Tregs suppresses the proliferation of CD8^+^ T cells to balance the immune response in a system by including cell cycle arrest or apoptosis. Therefore, we defined the corresponding rules to implement Treg-induced inhibition of CD8^+^ T cell proliferation. If a CD8^+^ T cell is in a certain phase of cycle, our rules would be paused once. In addition, a Treg cell may directly induce apoptosis of the adjacent CD8^+^ T cell. The selection of cell cycle arrest or apoptosis will be determined by dice rolling.

### Tissue scale

In the tissue scale of this HABM model, the secretion of SDF-1 from MICs and the diffusion of SDF-1 in the 3D ECM defined the dynamic 3D distribution of SDF-1 concentration. TGFβ can be secreted from both BMSC and MM, and the diffusion of TGFβ in the ECM also defined the dynamic 3D distribution of TGFβ, which involved in the regulation of the immune system. SDF-1 and TGFβ are uniformly initialized at the start with Dirichlet boundary. The drug concentration determined the chemical microenvironment in bone marrow. In addition, the tissue stiffness defined by BMSC contraction determined the biophysical microenvironment.

### ODE-based models of intracellular signaling response

### ODE system of SDF-1-triggerred BMSC stiffness

Each BMSC agent has encapsulated signaling transduction functions to determine its biomechanical phenotype switch. In response to the in-silu relative SDF-1 concentration, the binding of SDF-1 to the CXCR4 results a SDF-1/CXCR4 signaling pathway activation, which regulates the local stiffness. To describe this dynamic process, we developed an ODE-based dynamic model to predict the biomechanical properties of BMSCs based on the local concentration of SDF-1.

The ODE system of SDF-1/CXCR4 signaling pathways has the following form:

$$\frac{d\left[PI3K\right]}{dt}=k_{1}\frac{\left[SDF1\right]}{H_{1}+\left[SDF1\right]}-d_{1}\left[PI3K\right]$$ (1)

$$\frac{d\left[MEK\right]}{dt}=k_{2}\frac{\left[SDF1\right]}{H_{2}+\left[SDF1\right]}-d_{2}\left[MEK\right]$$ (2)

$$\frac{d\left[FAK\right]}{dt}=k_{3}\frac{\left[PI3K\right]}{H_{3}+\left[PI3K\right]}-d_{3}\left[FAK\right]$$ (3)

$$\frac{d\left[RhoA\right]}{dt}=k_{4}\frac{\left[PI3K\right]}{H_{4}+\left[PI3K\right]}+k_{5}\frac{\left[MEK\right]}{H_{5}+\left[MEK\right]}-d_{4}\left[RhoA\right]$$ (4)

$$\frac{d\left[ERK\right]}{dt}=k_{6}\frac{\left[MEK\right]}{H_{6}+\left[MEK\right]}-d_{5}\left[ERK\right]$$ (5)

$$\frac{d\left[MYL2\right]}{dt}=k_{7}\frac{\left[RhoA\right]}{H_{7}+\left[RhoA\right]}+k_{8}\frac{\left[ERK\right]}{H_{8}+\left[ERK\right]}-d_{6}\left[MYL2\right]$$ (6)

$$\frac{d\left[stiffness\right]}{dt}=k_{9}\frac{\left[FAK\right]}{H_{9}+\left[FAK\right]}+k_{10}\frac{MYL2}{H_{10}+\left[MYL2\right]}-d_{7}\left[stiffness\right]$$ (7)

As mentioned above, our phosphor-proteomics data covered the key signaling proteins (pMEK, pFAK, pRhoA, pERK, and pMYL2), which were involved in this SDF-1/CXCR4 signaling network. The value of the unique input node for SDF-1 is a conditional variable, which is un-changed from 0 to 60 min. The node “stiffness” is the output variable of this ODE system. Choi *et al* reported that the average stiffness of myeloma BMSC will increase from 400 pa to 530pa after treatment with SDF-1 (100 ng/ml) *in vitro*. However, the real concentration of local SDF-1 in bone marrow is lower; therefore, we considered that 530pa is the maximum output value in our ODE system at 60 min after SDF-1 stimulation. All above parameters involved in this ODE system were estimated by optimizing formula (8) via GA algorithm:

$$\Theta^{*}=\underset{\Theta }{\arg\;\min}\sum_{i\in I1,t\in T1}|X_{i}^{t}-{\hat{X}}_{i}^{t}(\Theta )|$$ (8)

Where $X_{i}^{t}$ and ${\hat{X}}_{i}^{t}(\Theta )$ denote the measurement from the experiments and the theoretical results obtained from ODEs model of protein *i* at time point *t*. The parameter vector $\Theta =\{k_{1},\;H_{1},\;d_{1},\ldots .,k_{10},H_{10},\;d_{7}\}$ in above formulas (1–7) can be obtained by formula (8). The set *I*1is the indexes of observed proteins in the signaling network of BMSCs, and time series set *T*1 = {0,5 min, 10 min, 15 min, 60 min} includes all the time points in experimental data (Table S2 in Supplementary Data File). Supplementary Table S2 represents the estimated values of all parameters. The fitting accuracy of the predicted and measured values of key proteins is shown in Supplementary Figure S7.

### ODE system of MIC adhesion and survival

The adhesion and survival of MIC agents were also simulated using an ODE model. As the population data shown in Supplementary Figures S3–S4, we can see that (1) increase of stiffness promotes the survival rate of MIC cells; (2) MICs tend to adhere on the stiffer BMSCs rather than the softer ones or non-BMSC positions. Here, we assumed that each cell has the same probabilities for survival and adhension as the total population. Therefore, we developed an ODE dynamic model to predict the probabilities of survival and adhesion of MIC cell based on the local stiffness. The maximal value of input node “stiffness” was 530pa. Adhesion and survival rate of MICs were both predicted at 24 and 96 hours. The states (at 0.5 and 1 hours) of other proteins involved in this signaling network were normalized against its expression in 0 hour level. The ODE system has the following form:

$$\frac{d\left[FAK\right]}{dt}=k_{1}\frac{\left[stiffness\right]}{H_{1}+\left[stiffness\right]}-d_{1}\left[FAK\right]$$ (9)

$$\frac{d\left[Rac\right]}{dt}=k_{2}\frac{\left[FAK\right]}{H_{2}+\left[FAK\right]}+k_{3}\frac{\left[PI3K\right]}{H_{3}+\left[PI3K\right]}-d_{2}\left[Rac\right]$$ (10)

$$\frac{d\left[PI3K\right]}{dt}=k_{4}\frac{\left[FAK\right]}{H_{4}+\left[FAK\right]}-d_{3}\left[PI3K\right]$$ (11)

$$\frac{d\left[AKT\right]}{dt}=k_{5}\frac{\left[PI3K\right]}{H_{5}+\left[PI3K\right]}-d_{4}\left[AKT\right]$$ (12)

$$\frac{d\left[JNK\right]}{dt}=k_{6}\frac{\left[Rac\right]}{H_{6}+\left[Rac\right]}-d_{5}\left[JNK\right]$$ (13)

$$\frac{d\left[cJUN\right]}{dt}=k_{7}\frac{\left[JNK\right]}{H_{7}+\left[JNK\right]}-d_{6}\left[cJUN\right]$$ (14)

$$\frac{d[NFKB]}{dt}=k_{8}\frac{\left[Rac\right]}{H_{8}+[Rac]}+k_{9}\frac{\left[AKT\right]}{H_{9}+[AKT]}-d_{7}[NFKB]$$ (15)

$$\frac{d[Adhesion]}{dt}=k_{10}\frac{\left[cJUN\right]}{H_{10}+[cJUN]}-d_{8}[Adhesion]$$ (16)

$$\frac{d[Survial]}{dt}=k_{11}\frac{\left[NFKKB\right]}{H_{11}+[NFKB]}-K_{BTZ}\frac{D_{1}}{H_{BTZ}+D_{1}}-d_{9}[Survival]$$ (17)

All above parameters involved in this ODE system were estimated by optimizing formula (8) via GA algorithm.

$${ϒ}^{*}=\underset{ϒ}{\arg\;\min}\sum_{i\in I2,t\in T2}\left|Y_{i}^{t}-{\hat{Y}}^{t}{}_{i}(ϒ)\right|$$ (18)

Where *Y*_i_ and *Y*_i_ denote the measurement from the experiments and the theoretical results obtained from ODEs model of protein *i* at time point *t*. The parameter vector in above formula (9–17) can be obtained by formula (18). The set *I2*is the indexes of observed proteins in the signaling network of MICs, and includes all the time points in experimental data (Table S1 in Supplementary Data File). Supplementary Table S3 represents the estimated values of all parameters. The prediction of growth and drug resistance of SP U226 cells with different stiffness were shown in Supplementary Figure S3. Similarly, the adhesion of SP U226 cells towards different stiffness was inferred by ODEs (Supplementary Figure S4).

### Using hill functions in the intracellular scale

Except the ODE systems were applied to model the intracellular signaling networks in BMSC and MIC cells (Figure 2), Hill functions were used to simulate the signal transduction of other cells and further determine the cell behaviors (Supplementary Text).

### Stem cell fate determination

Once a MIC decided to enter cell cycle, it either generates two daughter cells, known as self-renewal, or to two MM cells, known as differentiation. The probability of cell fate was determined by stiffness-triggered MIC proliferation pathways via hill function, and the decision of each MIC cell was also realized by die casting simulation as mentioned above.

### Proliferation fates of MM

The fates of intermediate cell agents were determined by the probabilities of proliferation, which reflected the effects of cell neighborhood characters such as stiffness and cytokine concentration, and the current cell cycle status. When maximum renewal limit reached, a MM cell will die. According to the myeloma initiating cell hypothesis, only MIC can self-renew and proliferate without limits, however, the defined constant LGN is the maximum passage number of MM cells.

### Proliferation and survival fates of CD8^+^ T cells and Treg

The proliferation rate of CD8^+^ T cell depends on Treg population and the local concentration of TGFβ, which was also simulated by Hill Function. The proliferation and survival fates of a Treg agent were both determined by the local concentration of TGFβ via Hill Functions. When an immunomodulatory drug, such as LEN, was applied to treat MM/MIC, it would lead to a suppression of Tregs and stimulation of CD8^+^ T cells prolifeartion.

### Model implementation

The main components of ABM model were designed using the conception of “Object-Oriented Programming” and were achieved with C++. The ODEs modules of intracellular signaling pathways (in BMSC and MIC) were implemented by Fortran ODE Solver (DLSODE [42]) and were called in the ABM model. The proposed model was debugged and implemented under Linux environment on the platform of Texas Advanced Computing Center (TACC). All of the parameters in ABM model were tuned after running the system 200 times to fit the training data.

## SUPPLEMENTARY MATERIALS FIGURES AND TABLES







## Abbreviations

BM: Bone Marrow
MM: Multiple Myeloma
ABM: Agent-based Model
ODE: Ordinary Differential Equation
HABM: Hybrid multi-scale Agent-based Model
MIC: Myeloma Initiating Cell
BTZ: Bortezomib
LEN: Lenalidomide
Thal: Thalidomide
