# Supplementary material for: Predicting the impact of combined therapies on myeloma cell growth using a hybrid multi-scale agent-based model
Source: Oncotarget. 2016 Dec 9;8(5):7647–65. doi: 10.18632/oncotarget.13831 (PMC5352350; doi:10.18632/oncotarget.13831)
Supplement: Supplementary file 1 [file oncotarget-08-7647-s001.pdf]

Predicting the impact of combined therapies on myeloma cell growth using a hybrid multi-scale agent-based model

Supplementary Materials

Model development

In this study, we defined five types of agents in the HABM model to represent BMSC, MIC, MM, $CD8^+$, and Treg. We initialized the tumor extracellular matrix (ECM) as a 100*100*100 3D matrix. BMSC cells were simulated as edges of 3D rectangular framework embedded into the ECM. The length of each edge was four grids (40 μm). At the beginning, the mixed population of MIC, MM, $CD8^+$, and Treg, were initialized in the center of the 3D ECM as a sphere. The numbers of each type of cell are 100 (MIC), 100(MM), 20($CD8^+$), and 5(Treg). The proposed multi-scale modeling consisted of three scales: intracellular, intercellular and tissue scales, which were illustrated in the section of "Materials and Methods", and described in details in the following sections.

Stochastic simulation of cell behaviors

The Markov Chain Monte Carlo approach was used to simulate cell behaviors of each individual cell. As shown in Figure 10, cell behaviors were simulated by probability-based rule implementation. A cell sensed the hints of its neighborhood such as stiffness, SDF-1 and TGFβ level, and drug doses, processed them with signaling pathways (via OED systems or Hill functions), and outputted the changes of probabilities of corresponding cell behaviors including cell proliferation rate, survival (apoptosis) rate, differentiation ratio, migration rate, and cytokine secretion rate. Cell decision was then determined by rolling a dice and compared with the probability of a given cell behavior. Cell behaviors in turn updated the properties of its niches. Details of each cell behavior for each type of cell agent as well as the corresponding rule was introduced in the following sections.

Intracellular scale

Each BMSC agent determines its biomechanical phenotype via the ODE system of SDF-1/CXCR4 signaling pathway (see Materials and Methods). Each grid point of the BMSC represented a section of the BMSC reticular. Each BMSC agent senses the local concentration of SDF-1, then activates the SDF-1/CXCR4 pathways, and finally output the stiffness.

Similarly, the oncogenic effect of the niche stiffness on the proliferation rates of myeloma initiating cells (MIC) and mature multiple myeloma (MM) were described with a Hill function (Eq.1).

$$P^l_{prol} = P^l_0 + P^l_{pathway}\frac{(\frac{E-E_0}{K_E})^2}{1+(\frac{E-E_0}{K_E})^2} \tag{1}$$

Where P^l_{prol}, $l = 1, 2$ were the proliferation rates of MIC, and MM, respectively. P^l_0 denotes the initial proliferation rate, $P^l_{pathway}$ is the maximal proliferation rate of the embedded signaling pathway in BMSC cells. K_E, E, and E_0 were the maximal stiffness, local stiffness, and base stiffness value. We assumed that the contraction of a BMSC section altered the stiffness of its local extracellular matrix, and thus defined the stiffness a myeloma cell sensed E, as the maximal stiffness of its neighbor BMSC sections within 3 grid distance.

The proliferation rate of $CD8^+$ T cell was not only regulated by local concentration of TGFβ and the dose of Lenalidomide, but also was affected by the population of Tregs (Eq. 2).

$$Prol_{CD8+} = Prol^0_{CD8+} + Prol^1_{CD8+} * \left(1+\frac{\left(\frac{PL}{K_L}\right)^n}{1+\left(\frac{PL}{K_L}\right)^n}\right) * \left(\frac{\left(\frac{R}{K_r}\right)^m}{1+\left(\frac{R}{K_r}\right)^m}\right) * \left(\frac{1}{1+\left(\frac{T_{ijk}}{K_T}\right)^2}\right) \tag{2}$$

Where $Prol^0_{CD8+}$ is the base proliferation rate of $CD8^+$ T cells. PL denotes the relative LEN dose and K_L the sensitivity of the $CD8^+$ T cells to LEN. T_{ijk} denotes the relative TGFβ concentration at the gird point (i, j, k); and K_T denotes the threshold TGF level to suppress $CD8^+$ T cells. The effect of Tregs on $CD8^+$ was described with R, which determined by the population ratio of Treg against $CD8^+$. Based on the Haart's experimental data [1], we inferred the formulation of function R according to different ratios of Treg to $CD8^+$ (Eq. 3).

$$R = 0.82 - 3.03 * \frac{ratio - 0.0659}{3.5609 + (ratio - 0.0659)} \tag{3}$$

$$ratio = \frac{Tregs}{CD8+} \tag{4}$$

According to formula (3–4), we can clearly know that increase of Treg population will suppress the proliferation of $CD8^+$.

Finally, the proliferation rate of Treg cell was defined as Eq. 5.

$$Prol_{Treg} = Prol_{Treg}^{0} + Prol_{Treg}^{1} * \left(1 + \frac{\left(\frac{T_{ijk}}{K_T}\right)^2}{1+\left(\frac{T_{ijk}}{K_T}\right)^2} \right) * \left(\frac{1}{1+\left(\frac{PL}{K_L}\right)^2} \right) \tag{5}$$

Where $Prol_{Treg}^{0}$ is the base proliferation rate of regulatory T cells (Treg). Formula (5) indicates that the production of Tregs can be promoted by TGFβ and suppressed by IMiD drug (such as, LEN).

Cell decision-making process was defined by agent rules, and the stochastic feature of the decision of an individual cell was realized by die casting simulation. Here, we used the cell decision-making of entering cell cycle as an example to elaborate the algorithm. Cell proliferation rules were defined according to proliferation rates as probabilities of two cell decisions with a die $C_{rand} \in [0,1]$.

$$\begin{cases} C_{rand} \in [0, P_{prol}) & \textit{cell cycle } \text{ON} \\ C_{rand} \in [P_{prol}, 1] & \textit{cell cycle } \text{OFF} \end{cases} \tag{6}$$

If the die fell into the interval $[0, P_{prol}]$, the cell entered cell cycle and started to proliferate; otherwise, stayed quiescent. Four cell cycle phases [2, 3] (G0/G1, S, G2, and M) were defined. Cells kept migrating during the first three phases, while tried to find a location to divide after entering the M phase, which was defined in the following sections. The duration of stage G0/G1 was determined by the Monte Carlo simulation (die casting) process, the S phase lasted for 20 hours (10 time steps), and the G2 phase would not exceed 10 hours (5 time steps).

Stem cell fate determination

Once a MIC decided to enter cell cycle, its fates were further determined according to its microenvironment. A MIC either generates two MIC daughter cells, known as self-renewal, or to two MM cells, known as differentiation. The probability of each fate was denoted as r_r. Rules for MIC fate determination were represented in Eq.7:

$$\begin{cases} C_{rand} \in [0, r_r) & \text{self renewal} \\ C_{rand} \in [r_r, 1] & \text{differentiation} \end{cases} \tag{7}$$

Where $r_r = r_{r,0} + \Delta r$. Δr describes the effects of stiffness-trigged MIC signaling pathway on cell fates, and was determined by Eq.8:

$$\Delta r = r_{pathway} \frac{\left(\frac{E-E_0}{K_{RE}}\right)^2}{1+\left(\frac{E-E_0}{K_{RE}}\right)^2} \tag{8}$$

Where denotes the signaling pathway activation threshold.

Proliferation fate of MM agents. The fates of MM cell agents were determined by their passage age (Eq. 9).

$$\text{MM} \rightarrow \begin{cases} \text{2MM} & \text{generation number} \le \text{LGN} \\ \text{Dead} & \text{generation number} > \text{LGN} \end{cases} \tag{9}$$

Intercellular scale

Each type of cells would proliferate, migrate, being quiescent, or undergo death, which were described in the intercellular scale.

Migration

A non-M-phase cell at position p would migrate if it would find free space nearby. Except BMSC agents, other four types of cell agents can migrate from one place to another free space. MICs and MMs prefer to migrate towards the stiffer BMSCs. Once they attached on BMSC sections, they prefer to stay there rather than move to non-BMSC position (Supplementary Figure S2). For the $CD8^+$ T cells, they tried to migrate towards Myeloma cells (MICs and MMs) and to induce the lysis of tumor cells. As to Tregs, they prefer to migrate to $CD8^+$ T cell population, and to induce the cell cycle arrest or apoptosis of the effector cells.

Now, we firstly introduce the migration of MIC. In this study, if a MIC cell will migrate depends on what's its current position. If a MIC cell is not attaching on BMSC, it will definitely implement the procedure of migration; otherwise, we firstly calculate the adhesion rate (P_{adh}) of MIC with local stiffness via ODE system, then make dice rolling to generate a random value *md* and to determine if it will migrate to another place ($P_{adh} < md$). According to Supplementary Figure S2, we can find that MIC prefers to continuously stay on BMSC rather than move to non-BMSC position. The migration was defined in the following rules. All the unoccupied positions within a radius r_{max} (Eq. 10)

$$r_{max} = \left\lfloor \frac{2(1+4D\Delta t)}{10} \right\rfloor + 1 \tag{10}$$

Were listed as candidates, where D was the basic migration speed index, Δt is the time step (2hours). All candidate locations were ranked as Eq. 11.

$$R_l = p(r_l) \, . \, \max\left(C_l^n\right) . C_{CTL} \, . \, V_l \tag{11}$$

R_l was the ranking score of each candidate position P_{ijk}. Variable r_l is the distance from the candidate location to the original position P_{ijk}, and the subscript *ijk* is the coordinates of the candidate locations. $p_{(rl)}$ is the visiting chance of P_{ijk} (Eq. 12).

$$p(r_l) = \frac{1}{4\pi D\Delta t} \exp\left(\frac{-r_l^2}{4\pi D\Delta t} \right) \tag{12}$$

$C_l^n, n = 1,\ldots,6$ were the preference of neighbor at P_{ijk}. The position P_{ijk} had six immediate neighbors. The value of C_l^n was defined by Eq. 13.

$$C_l^n \rightarrow \begin{cases} F_E\left(E_{xyz}^n\right) & \text{if the n-th neighbour is BMSC} \\ \frac{1}{4} & \text{otherwise} \end{cases} \quad (13)$$

Where

$$F_E\left(E_{xyz}^n\right) = F_{E,0} + F_{E,max}\frac{\left(\frac{E_{xyz}^n - E_0}{K_{FE}}\right)^2}{1+\left(\frac{E_{xyz}^n - E_0}{K_{FE}}\right)^2} \quad (14)$$

The coefficients $F_{E,0}$ and $F_{E,max}$ were the weights of Eq. 14 which described the degree of preference of an MIC attaching to an unprimed BMSC and the increase of such preference to a primed MBMSC. E_{xyz}^n was the stiffness at P_{ijk}'s n-th neighbor with the exact coordinate (x,y,z). E_0 was the minimal sensed stiffness and E,max the maximal. $K_{FE} = \frac{E_{max} - E_0}{2}$ Variable C_{CTL} denotes if P_{ijk}' exists a neighbor as CD8+ T cell. If yes, the value of C_{CTL} is small to decrease the chances of MICs migration to CD8+; otherwise, C_{CTL} is large (Eq. 15).

$$C_{CTL} \rightarrow \begin{cases} \frac{1}{4} & \text{at least a neighbor is CD8} \\ 1 & \text{otherwise} \end{cases} \quad (15)$$

Variable V_l was defined as Eq. 16.

$$V_l = \begin{cases} \frac{1}{8} & \text{P}_{\text{ijk}} \text{ has } 5-6 \text{ neighbor cells} \\ \frac{1}{4} & \text{P}_{\text{ijk}} \text{ has } 3-4 \text{ neighbor cells} \\ 1 & \text{P}_{\text{ijk}} \text{ has } 1-2 \text{ neighbor cells} \\ \frac{1}{16} & \text{P}_{\text{ijk}} \text{ has no neighobr cells} \end{cases} \quad (16)$$

Eq. 16 represents that myeloma cells try to avoid loneliness as well as crowdedness, though they are not as sensitive as solid tumor cells are. Ranks of all candidates were normalized so that the sum of all normalized ranks is 1 (Eq. 17).

$$\hat{R}_l = \frac{R_l}{\sum_l R_l} \quad (17)$$

To facilitate die-casting, all normalized ranks were incorporated to form a scale S, in which each candidate is corresponding to a range .

$$S = \left\{S_l : S_l = \left\lfloor \sum_{m<l} \hat{R}_m, \hat{R}_l + \sum_{m<l} \hat{R}_m \right\rfloor\right\} \quad (18)$$

The dice casting generates a random value $d \in [0,1)$. If d falls in S_l, the position P_{ijk} is chosen as the next migration stop.

For the MM, its migration rules were very similar as MIC except the definition of variable C_l^n. Here, C_l^n was set to 1 if the n-th neighbor of P_{ijk} was BMSC (Eq. 19).

$$C_l^n \rightarrow \begin{cases} 1 & \text{if the } n-\text{th neighbor is BMSC} \\ \frac{1}{4} & \text{otherwise} \end{cases} \quad (19)$$

For the CD8+ T cell, it will migrate towards MIC or MM rather than empty position and BMSC. The CD8-mediated lysis of myeloma cell might probably occur the positions where are not BMSC. In other word, myeloma cell adhere to BMSC will reduce the probability of apoptosis and escape from immune system [1]. The candidate rank was calculated with Eq.20.

$$R_l = p(r_l) \,.\, \max\left(C_l^n\right) .V_l \quad (20)$$

In Eq.20, the values of $p(r_l)$ and V_l are also calculated by Eq.12 and Eq.16. The value of C_l^n for CD8+ T cell was defined by Eq.21.

$$C_l^n \rightarrow \begin{cases} 1 & \text{if the } n-\text{th neighbor is MIC/MM} \\ \frac{1}{2} & \text{otherwise} \end{cases} \quad (21)$$

As to the regulatory T cell (Treg), it will migrate towards CD8+ T cell and try to suppress the proliferation of CTL. The candidate rank was also calculated with Eq.20. In Eq.20, the values of $p(r_l)$ and V_l are also calculated by Eq.12 and Eq.16. The value of C_l^n for Treg was defined by Eq.22.

$$C_l^n \rightarrow \begin{cases} 1 & \text{if the } n-\text{th neighbor is CD8+} \\ \frac{1}{2} & \text{otherwise} \end{cases} \quad (22)$$

CD8+ mediated Lysis of myeloma cells

During the migration of CD8+ T cells, they moved towards to target cells (MICs or MMs) and tried to kill them. When a CD8+ T cell located at a position where there was a myeloma cell, the target cell was labeled with a status variable and will put into apoptosis list in the next time step.

Suppression effect of Tregs on CD8+

When regulatory T cells were activated, they would sharply colon themselves. Tregs would move towards CD8+ T cells and tried to suppress the generation of CD8+ T cells by inducing cell cycle arrest or direct apoptosis.

Apoptosis

At each time step, if the apoptosis rate of the cell (MIC, MM, CD8+, and Treg) was lesser than the threshold; all four types of cells in this system would start apoptosis. Cell took 10 time steps to finish apoptosis and was then absorbed. As we discussed in this study, cytotoxic drug BTZ increased the apoptosis rate of cancer cell as Eq. 23 (for MIC) and Eq.24 (for MM).

$$Apop_{MIC} = Apop_{MIC}^{0} + Apop_{MIC}^{1} * \left(1 - Sur_{MIC}^{BTZ} * Sur_{CD8}\right) \quad (23)$$

According to Eq.23, we can see that the apoptosis of MIC cell are regulated by two factors: (1) BTZ treatment; (2) CD8+ mediated lysis. They were defined with Eq. 24–25.

$$Sur_{MIC}^{BTZ} = \frac{\left(\frac{S_a}{K_a}\right)^3}{1 + \left(\frac{S_a}{K_a}\right)^3} \quad (24)$$

$$Sur_{CD8} = \frac{\left(\frac{S_b}{K_b}\right)^4}{1 + \left(\frac{S_b}{K_b}\right)^4} \quad (25)$$

Where S_a is the ODE-inferred survival rate based on the local stiffness and relative concentration of BTZ. S_b is the CD8-induced survival ratio of myeloma cell [1]. As to MM cell, its apoptosis rate was defined as Eq. 26.

$$Apop_{MM} = Apop_{MM}^{0} + Apop_{MM}^{1} * \left(1 - Sur_{MM}^{BTZ} * Sur_{CD8}\right) \quad (26)$$

$$Sur_{MM}^{BTZ} = \frac{\left(\frac{S_c}{K_c}\right)^4}{1 + \left(\frac{S_c}{K_c}\right)^4} \quad (27)$$

Where S_c is the BTZ-induced survival ratio inferred from literature [4]. At last, the apoptosis rates of CD8+ and Treg were defined as Eq.28–29.

$$Apop_{CD8} = Apop_{CD8}^{0} + Apop_{CD8}^{1} * \left(1 + \frac{\frac{T_{ijk}}{K_T}}{1 + \frac{T_{ijk}}{K_T}}\right) \quad (28)$$

$$Apop_{Treg} = Apop_{Treg}^{0} + Apop_{Treg}^{1} * \left(1 + \frac{\left(\frac{LEN}{K_L}\right)^2}{1 + \left(\frac{LEN}{K_L}\right)^2}\right) * \left(\frac{1}{1 + \frac{T_{ijk}}{K_T}}\right) \quad (29)$$

Tissue scale

In the tissue scale of this HABM model, the secretion of SDF-1 (from MIC) and TGF (From both MM and BMSC) and the diffusion of them in the 3D ECM defined the dynamic 3D distribution of SDF-1 and TGFβ, which were both described as Eq.30.

$$S_{ijk}(t+1) = (1 - deg) * \left[S_{ijk}(t) * (1 - \mu) + \frac{\mu}{6}\sum_{i=1}^{6} S_{ijk}^{l}(t)\right] \quad (30)$$

$S_{ijk}(t+1)$ was the concentration of the factor on the location P_{ijk} in the time step t+1, and μ was the diffusion constant. $S_{ijk}^{l}(t), l = 1,2,\ldots,6$ were the factor's values of P_{ijk}'s six immediate neighbors in the current time step t. The constant *deg* represented the degrading rate.

REFERENCES

1. de Haart SJ, van de Donk NW, Minnema MC, Huang JH, Aarts-Riemens T, Bovenschen N, Yuan H, Groen RW, McMillin DW, Jakubikova J, et al. Accessory cells of the microenvironment protect multiple myeloma from T-cell cytotoxicity through cell adhesion-mediated immune resistance. Clin Cancer Res. 2013; 19:5591–5601.
2. Alarcon T, Byrne HM, Maini PK. A mathematical model of the effects of hypoxia on the cell-cycle of normal and cancer cells. J Theor Biol. 2004; 229:395–411.
3. Zhang L, Athale CA, Deisboeck TS. Development of a three-dimensional multiscale agent-based tumor model: simulating gene-protein interaction profiles, cell phenotypes and multicellular patterns in brain cancer. J Theor Biol. 2007; 244:96–107.
4. Campanella A, Santambrogio P, Fontana F, Frenquelli M, Cenci S, Marcatti M, Sitia R, Tonon G, Camaschella C. Iron increases the susceptibility of multiple myeloma cells to bortezomib. Haematologica. 2013; 98:971–979.
5. Choi DS, Stark DJ, Raphael RM, Wen J, Su J, Zhou X, Chang CC, Zu Y. SDF-1alpha stiffens myeloma bone marrow mesenchymal stromal cells through the activation of RhoA-ROCK-Myosin II. Int J Cancer. 2015; 136:E219–229.
6. Feng Y, Wen J, Mike P, Choi DS, Eshoa C, Shi ZZ, Zu Y, Chang CC. Bone marrow stromal cells from myeloma patients support the growth of myeloma stem cells. Stem Cells Dev. 2010; 19:1289–1296.
7. Feng YD, Ofek G, Choi DS, Wen JG, Hu J, Zhao H, Zu YL, Athanasiou KA, Chang CC. Unique biomechanical interactions between myeloma cells and bone marrow stroma cells. Prog Biophys Mol Bio. 2010; 103:148–156.
8. Su J, Zhang L, Zhang W, Choi DS, Wen J, Jiang B, Chang CC, Zhou X. Targeting the biophysical properties of the myeloma initiating cell niches: a pharmaceutical synergism analysis using multi-scale agent-based modeling. PloS One. 2014; 9:e85059.
9. Henry JY, Labarthe MC, Meyer B, Dasgupta P, Dalgleish AG, Galustian C: Enhanced cross-priming of naive CD8+ T cells by dendritic cells treated by the IMiDs(R) immunomodulatory compounds lenalidomide and pomalidomide. Immunology. 2013; 139:377–385.
10. Karel Fostier JC, Carlo Heirman, Joeri L. Aerts, Kris Thielemans, Rik Schots, and Brenda De Keersmaecker.: Immunomodulatory Drugs Restore Effector Cell Immune Functions In Myeloma Patients With Low Disease Burden After Autologous Stem Cell Transplantation. Blood. 2013; 122.
11. Wen J, Feng Y, Huang W, Chen H, Liao B, Rice L, Preti HA, Kamble RT, Zu Y, Ballon DJ, et al. Enhanced antimyeloma cytotoxicity by the combination of arsenic trioxide and bortezomib is further potentiated by p38 MAPK inhibition. Leuk Research. 2010; 34:85–92.

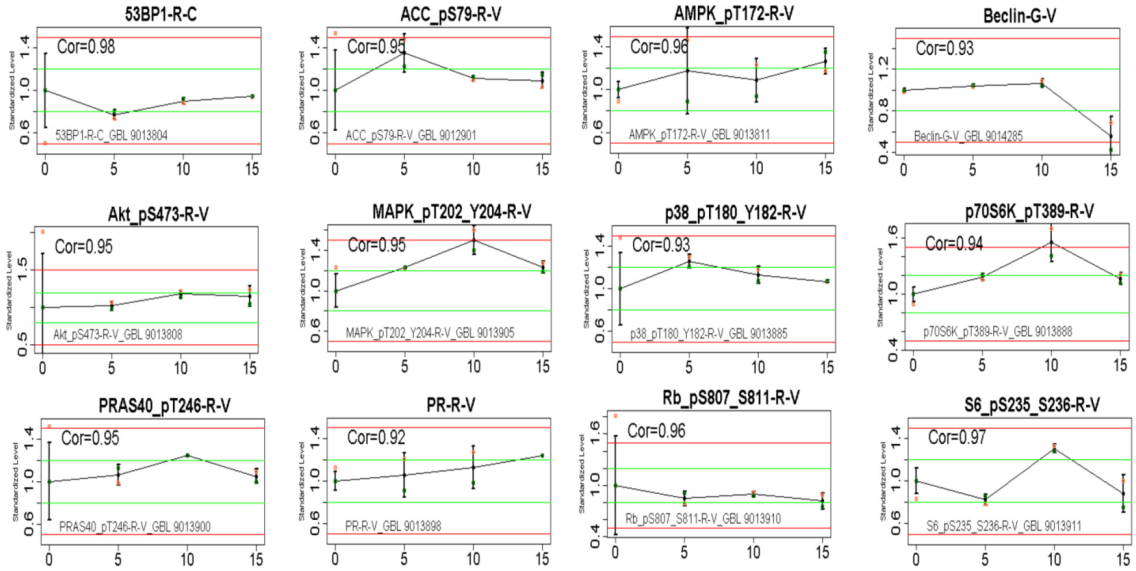

Supplementary Figure S1: Significant expressed proteins in the RPPA dataset of BMSCs.

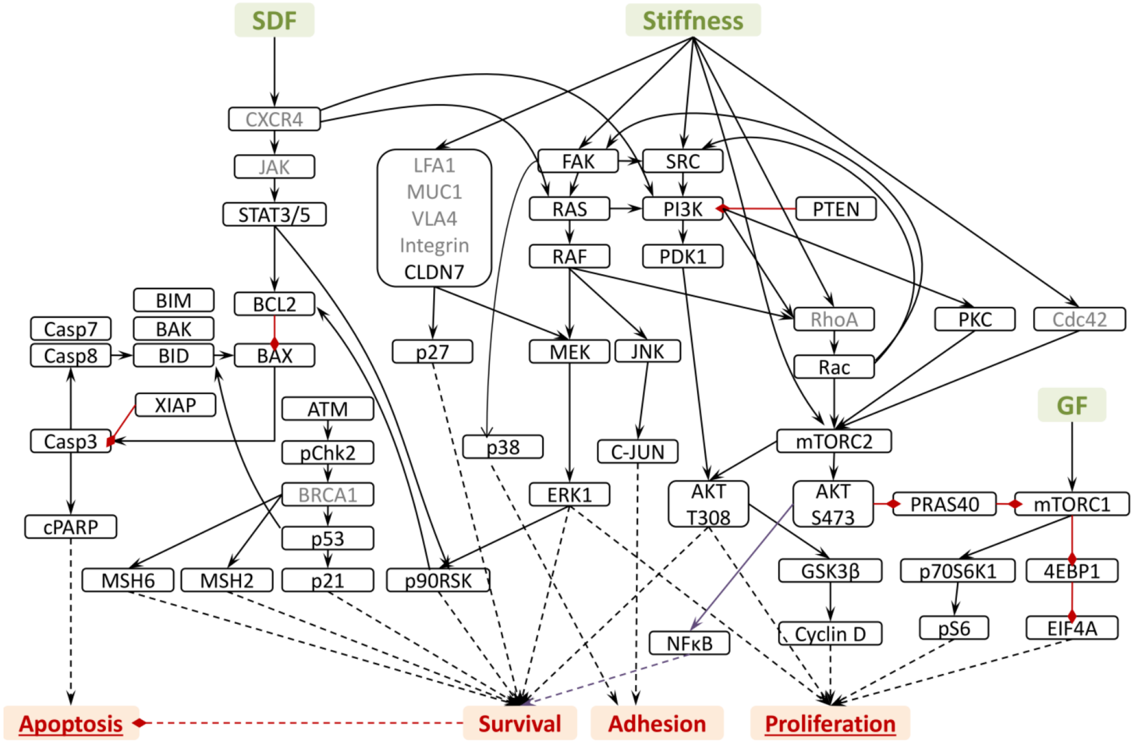

Supplementary Figure S2: Manully developed signaling pathway map of MIC based on RPPA data.

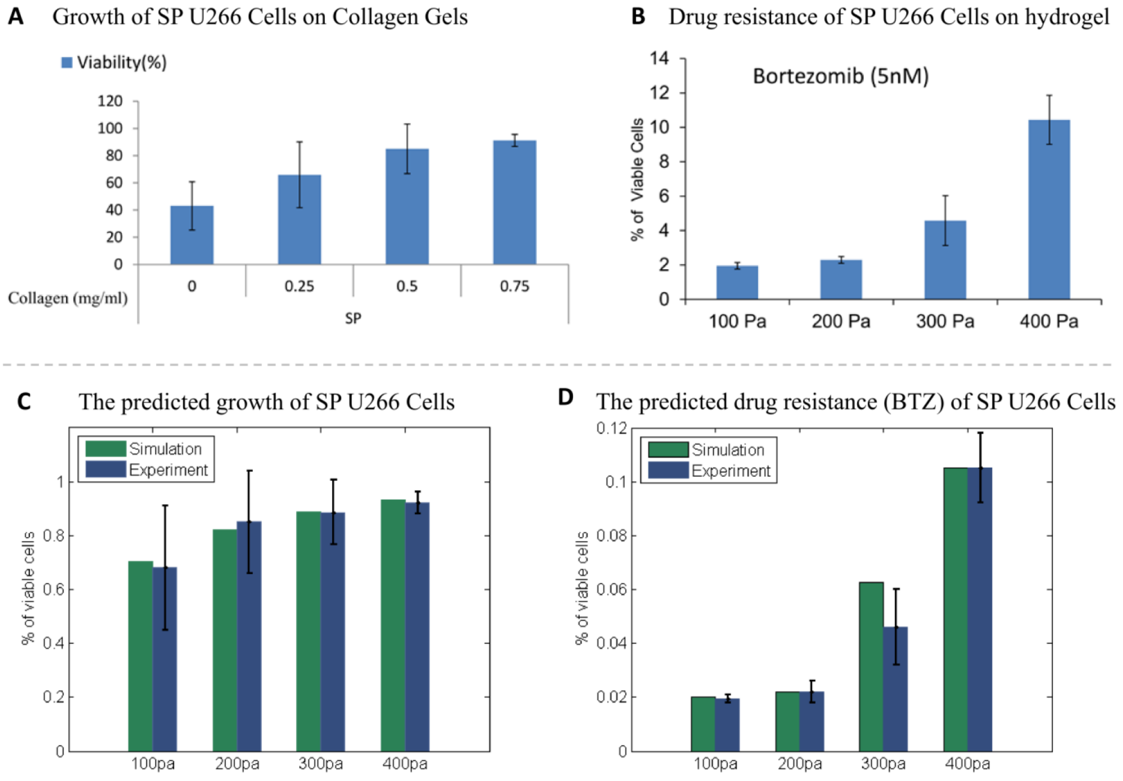

Supplementary Figure S3: Growth and Drug resistance of SP U266 Cells on Hydrogel and Collagen Gels and model prediction. (**A**) the cell viability of MIC cells on Collagen gels. (**B**) growth of MIC cells on hydrogel treated 6 days by Bortezomib. (**C–D**). The predicted cell viabilities of MIC cells with or without BTZ treatment.

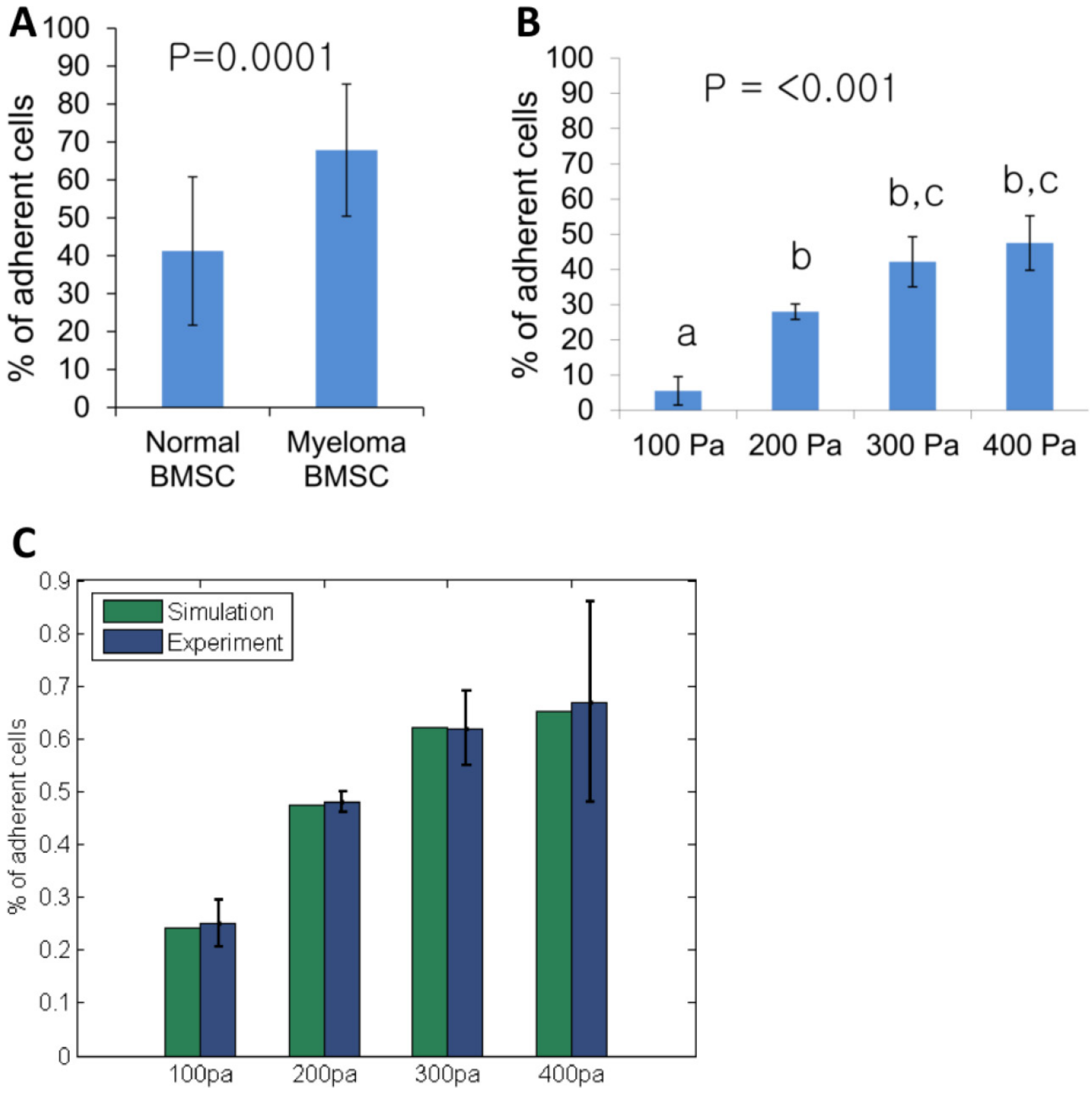

Supplementary Figure S4: The adhesion of MIC cells. (**A**) the difference of adhesion ability between Normal BMSC and Myeloma BMSC. (**B**) stiffness-induced effect on the adhesion of MICs. (**C**) The predicted adhesion ability of MICs on hydrogel with different stiffness levels.

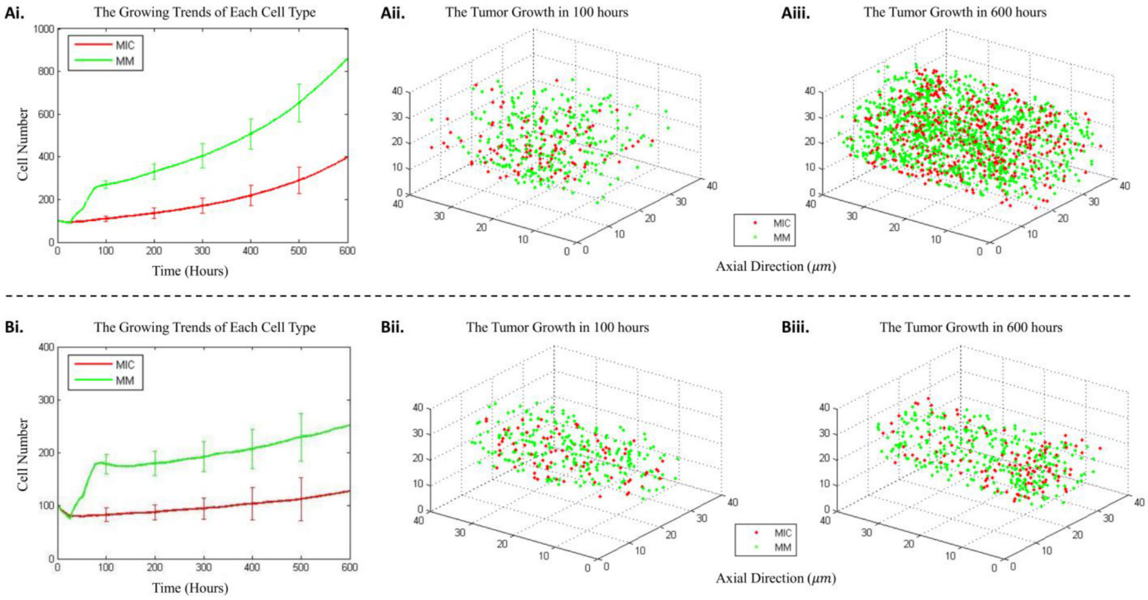

Supplementary Figure S5: The growth of myeloma in bone marrow with or without immune system.

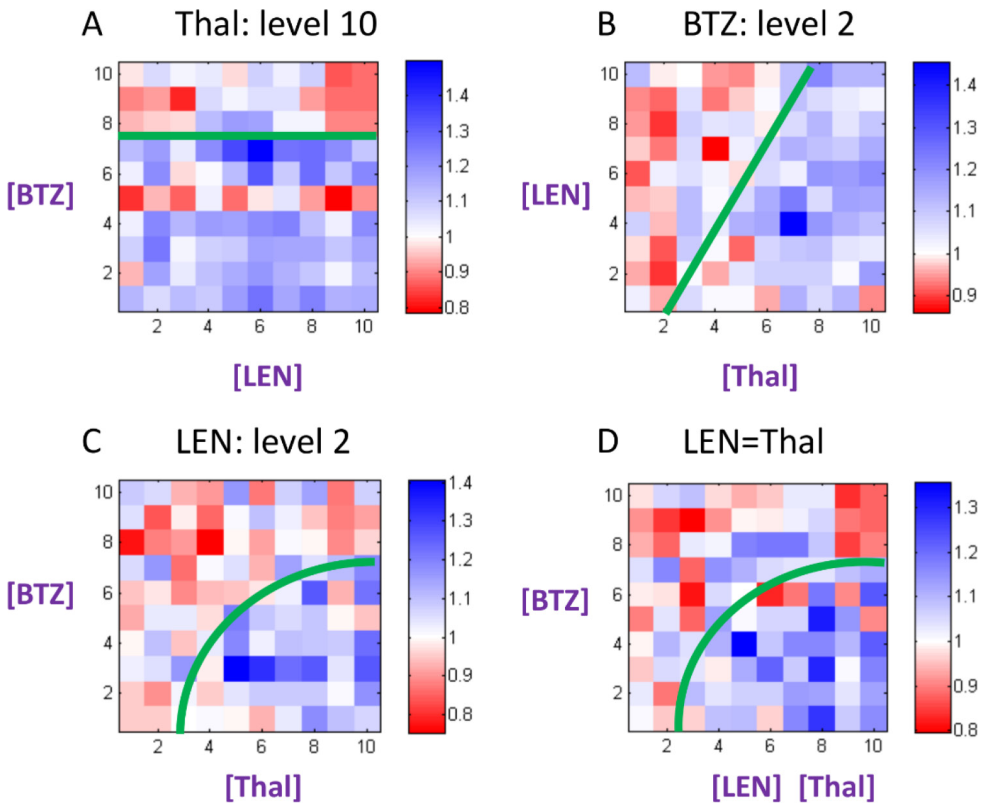

Supplementary Figure S6: Synergy effects of BTZ, LEN, and Thal on total myeloma growth in BMSC niches. Blue points indicate synergistic and red points represent antagonistic.

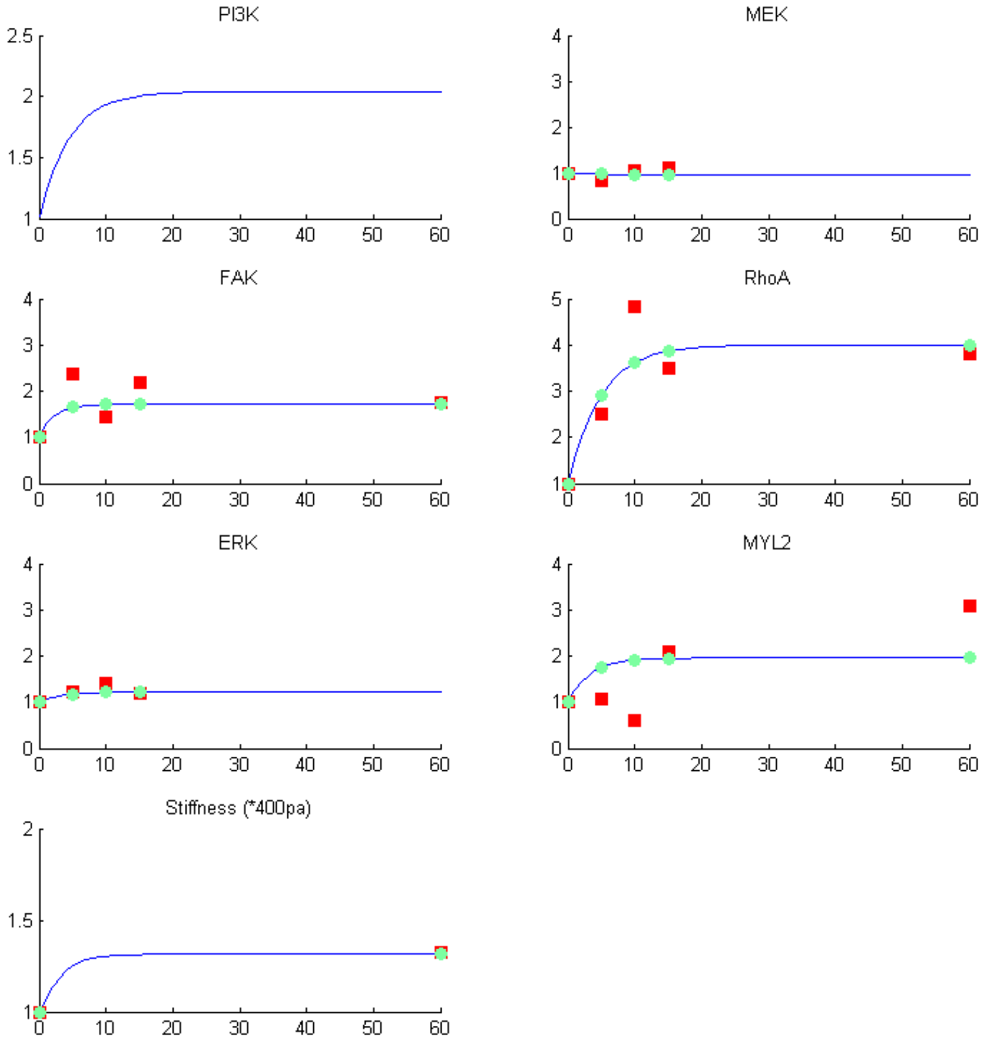

Supplementary Figure S7: The fitting curves of the ODE model of myeloma BMSC.

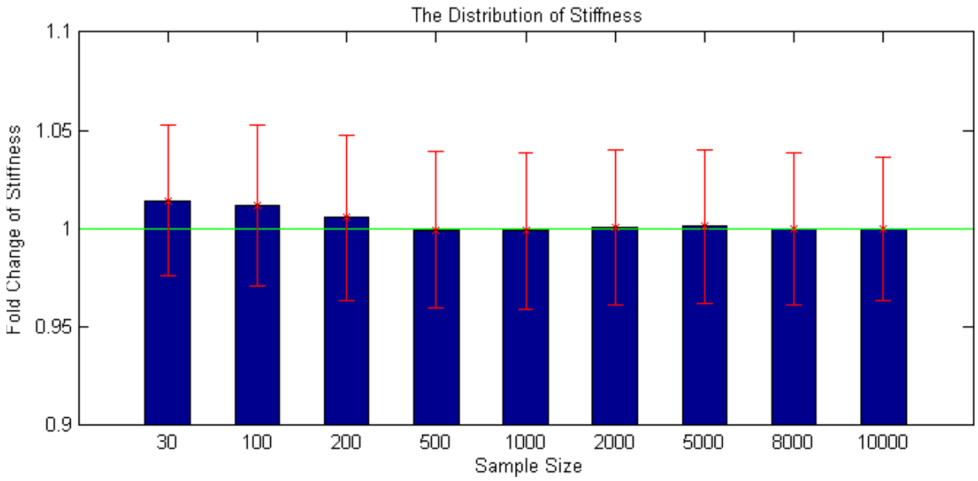

Supplementary Figure S8: The results of uncertainty analysis for the output variable stiffness in the ODE system in BMSC. The line with green color corresponds to the output of the baseline model (BMSC).

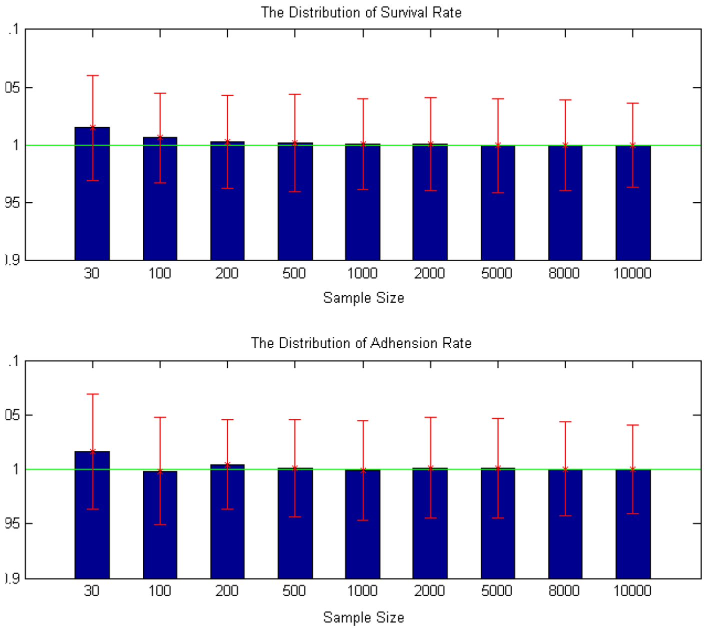

Supplementary Figure S9: The results of uncertainty analysis for the output variables (survival rate and adhesion rate) in the ODE system in MIC. Two lines with green color correspond to survival rate and adhesion rate of the baseline model (MIC).

Supplementary Table S1: The significant expressed proteins in MIC under condition of 400 pa to 100 pa

Antibody Name	400 Pa VS 100 Pa
HER2_pY1248	2.67403
IRS-1(V)	2.21406
HSP70(c)	2.209577
Pras40 pT246(V)	2.099551
BCL-2(V)(Mouse)	2.099497
P90RSK_pT359_S363(C)	2.085224
Shc	1.993025
IGFBP2(V)	1.757789
PARP cleaved	1.754373
ACC1(c)	1.634121

Supplementary Table S2: The estimated parameters in the ODE system of SDF1-triggerred BMSC stiffness

K_1	0.5609	H_1	0.2244	d_1	0.2246
K_2	0.5004	H_2	0.5020	d_2	0.3456
K_3	0.98	H_3	0.1	d_3	0.5441
K_4	0.8905	H_4	0.1902	d_4	0.2199
K_5	0.0910	H_5	0.3744	d_5	0.2101
K_6	0.7677	H_6	0.9960	d_6	0.5342
K_7	0.7731	H_7	0.9231	d_7	0.6791
K_8	0.6381	H_8	0.6379		
K_9	0.1729	H_9	0.1232		
K_{10}	0.9854	H_{10}	0.6788		

SupplementaryTable S3: The estimated parameters in the ODE system of MICs

K_1	0.5932	H_1	0.2164	d_1	0.1284
K_2	0.3480	H_2	0.8460	d_2	0.524
K_3	0.0310	H_3	0.9893	d_3	0.6183
K_4	0.8107	H_4	0.8363	d_4	0.1347
K_5	0.3256	H_5	0.0447	d_5	0.2110
K_6	0.4187	H_6	0.4953	d_6	0.6820
K_7	0.6662	H_7	0.1271	d_7	0.5872
K_8	0.7570	H_8	0.3366	d_8	0.3591
K_9	0.5238	H_9	0.1182	d_9	0.8035
K_{10}	0.3867	H_{10}	0.5586		
K_{11}	0.8945	H_{11}	0.3076		
K_{BZM}	0.7326	H_{BZM}	0.1023		

Supplementary Table S4: The parameters were used in ABM

Symbol	Variable	Initial value	Reference
E_0	Base stiffness of BMSC	250pa	[5]
K_E	Coefficient of Hill Function	$\frac{530-250}{2}$ pa	[5]
$P_0^{\,1}$	Initial proliferation rate of MIC cells	0.01	[6–8]
P_0^2	Initial proliferation rate of MM cells	0.065	
$P_pathway^1$ *	Effects of MIC stiffness pathway on proliferation	0.058	
$P_pathway^2$ *	Effects of MM stiffness pathway on proliferation	0	[8]
$Prol_{CD8+}^0$	Initial proliferation rate of CD8+ T cells	0.01	
$Prol_{(CD8+)}^1$ *	Effects of LEN dose, TGFβ concentration, and Treg population on proliferation of CD8+	0.02	
K_L*	Coefficient of Hill Function	0.47	[9]
K_r*	Coefficient of Hill Function	0.5	[10]
K_T*	Coefficient of Hill Function	0.5	
$Prol_{Treg}^0$ *	Initial proliferation rate of Treg	0.01	
$Prol_{Treg}^1$ *	Effects of LEN dose and TGFβ concentration on proliferation of CD8+	0.023	
r_{pathway}	Effects of MIC stiffness pathway on MIC self-renewal	0.15	[8]
D	Migration speed index	2 µm	[8]
$F_{\text{E},0}$*	Myeloma base preference to attached to MBSMC	0.5	[6, 7]
$F_{E,max}$*	Myeloma increased preference to attached to MBMSC	0.5	[6]
$Apop_{MIC}^0$	Initial apoptosis rate of MIC	0.005	[6–8, 11]
$Apop_{MIC}^1$	Effects of BTZ dose and CD8+ population on apoptosis of MIC	0.03	[1, 7, 8, 11]
$Apop_{MM}^0$	Initial apoptosis rate of MM	0.01	[6, 7, 11]
$Apop_{MM}^1$	Effects of BTZ dose and CD8+ population on apoptosis of MM	0.055	[1, 4]
K_a*	Coefficient of Hill Function	0.4	[6]
K_b*	Coefficient of Hill Function	0.5	[1]
K_c*	Coefficient of Hill Function	0.6	[4]
$Apop_{CD8+}^0$ *	Initial apoptosis rate of CD8+ T cells	0.01	
$Apop_{CD8+}^1$ *	Effects of TGFB concentration on apoptosis of CD8+ T cells	0.01	
$Apop_{Treg}^0$ *	Initial apoptosis rate of Tregs	0.01	
$Apop_{Treg}^1$ *	Effects of LEN dose and TGFβ concentration on apoptosis of Tregs	0.02	
Lamada_SDF1	Diffusion constant of SDF-1	0.612	[6, 7]
Lamada_TGFβ	Diffusion constant of TGFβ	0.44	[6, 7]

*: Indirectly inferred.
