# Supplementary material for: Predicting the impact of combined therapies on myeloma cell growth using a hybrid multi-scale agent-based model
Source: Oncotarget. 2016 Dec 9;8(5):7647–65. doi: 10.18632/oncotarget.13831 (PMC5352350; doi:10.18632/oncotarget.13831)
Supplement: Supplementary file 3 [file oncotarget-08-7647-s003.docx]

| **Supplementary Table S1: The significant expressed proteins in MIC under condition of 400 pa to 100 pa** | |
| --- | --- |
| **Antibody Name** | **400 Pa VS 100 Pa** |
| HER2_pY1248 | 2.67403 |
| IRS-1(V) | 2.21406 |
| HSP70(c) | 2.209577 |
| Pras40 pT246(V) | 2.099551 |
| BCL-2(V)(Mouse) | 2.099497 |
| P90RSK_pT359_S363(C) | 2.085224 |
| Shc | 1.993025 |
| IGFBP2(V) | 1.757789 |
| PARP cleaved | 1.754373 |
| ACC1(c) | 1.634121 |

| **Supplementary Table S2: The estimated parameters in the ODE system of SDF1-triggerred BMSC stiffness** | | | | | |
| --- | --- | --- | --- | --- | --- |
| K_1_ | 0.5609 | H_1_ | 0.2244 | d_1_ | 0.2246 |
| K_2_ | 0.5004 | H_2_ | 0.5020 | d_2_ | 0.3456 |
| K_3_ | 0.98 | H_3_ | 0.1 | d_3_ | 0.5441 |
| K_4_ | 0.8905 | H_4_ | 0.1902 | d_4_ | 0.2199 |
| K_5_ | 0.0910 | H_5_ | 0.3744 | d_5_ | 0.2101 |
| K_6_ | 0.7677 | H_6_ | 0.9960 | d_6_ | 0.5342 |
| K_7_ | 0.7731 | H_7_ | 0.9231 | d_7_ | 0.6791 |
| K_8_ | 0.6381 | H_8_ | 0.6379 |  |  |
| K_9_ | 0.1729 | H_9_ | 0.1232 |  |  |
| K_10_ | 0.9854 | H_10_ | 0.6788 |  |  |

| **SupplementaryTable S3: The estimated parameters in the ODE system of MICs** | | | | | |
| --- | --- | --- | --- | --- | --- |
| K_1_ | 0.5932 | H_1_ | 0.2164 | d_1_ | 0.1284 |
| K_2_ | 0.3480 | H_2_ | 0.8460 | d_2_ | 0.524 |
| K_3_ | 0.0310 | H_3_ | 0.9893 | d_3_ | 0.6183 |
| K_4_ | 0.8107 | H_4_ | 0.8363 | d_4_ | 0.1347 |
| K_5_ | 0.3256 | H_5_ | 0.0447 | d_5_ | 0.2110 |
| K_6_ | 0.4187 | H_6_ | 0.4953 | d_6_ | 0.6820 |
| K_7_ | 0.6662 | H_7_ | 0.1271 | d_7_ | 0.5872 |
| K_8_ | 0.7570 | H_8_ | 0.3366 | d_8_ | 0.3591 |
| K_9_ | 0.5238 | H_9_ | 0.1182 | d_9_ | 0.8035 |
| K_10_ | 0.3867 | H_10_ | 0.5586 |  |  |
| K_11_ | 0.8945 | H_11_ | 0.3076 |  |  |
| K_BZM_ | 0.7326 | H_BZM_ | 0.1023 |  |  |

**Supplementary Table S4: The parameters were used in ABM**

| **Symbol** | **Variable** | **Initial value** | **Reference** |
| --- | --- | --- | --- |
| *E_0_* | Base stiffness of BMSC | 250pa | [5] |
| *K_E_* | Coefficient of Hill Function | pa | [5] |
| *P*_0_^1^ | Initial proliferation rate of MIC cells | 0.01 | [6–8] |
|  | Initial proliferation rate of MM cells | 0.065 |  |
|  | Effects of MIC stiffness pathway on proliferation | 0.058 |  |
|  | Effects of MM stiffness pathway on proliferation | 0 | [8] |
|  | Initial proliferation rate of CD8+ T cells | 0.01 |  |
|  | Effects of LEN dose, TGFβ concentration, and Treg population on proliferation of CD8+ | 0.02 |  |
| *K_L_** | Coefficient of Hill Function | 0.47 | [9] |
| *K_r_** | Coefficient of Hill Function | 0.5 | [10] |
| *K_T_** | Coefficient of Hill Function | 0.5 |  |
|  | Initial proliferation rate of Treg | 0.01 |  |
|  | Effects of LEN dose and TGFβ concentration on proliferation of CD8+ | 0.023 |  |
| *r*_pathway_ | Effects of MIC stiffness pathway on MIC self-renewal | 0.15 | [8] |
| *D* | Migration speed index | 2 μm | [8] |
| *F*_E,0_*** | Myeloma base preference to attached to MBSMC | 0.5 | [6, 7] |
| *F_E,max_** | Myeloma increased preference to attached to MBMSC | 0.5 | [6] |
|  | Initial apoptosis rate of MIC | 0.005 | [6–8, 11] |
|  | Effects of BTZ dose and CD8+ population on apoptosis of MIC | 0.03 | [1, 7, 8, 11] |
|  | Initial apoptosis rate of MM | 0.01 | [6, 7, 11] |
|  | Effects of BTZ dose and CD8+ population on apoptosis of MM | 0.055 | [1, 4] |
| *K_a_** | Coefficient of Hill Function | 0.4 | [6] |
| *K_b_** | Coefficient of Hill Function | 0.5 | [1] |
| *K_c_** | Coefficient of Hill Function | 0.6 | [4] |
|  | Initial apoptosis rate of CD8+ T cells | 0.01 |  |
|  | Effects of TGFB concentration on apoptosis of CD8+ T cells | 0.01 |  |
|  | Initial apoptosis rate of Tregs | 0.01 |  |
|  | Effects of LEN dose and TGFβ concentration on apoptosis of Tregs | 0.02 |  |
| Lamada_SDF1 | Diffusion constant of SDF-1 | 0.612 | [6, 7] |
| Lamada_TGFβ | Diffusion constant of TGFβ | 0.44 | [6, 7] |

*: Indirectly inferred.
